# Transcriptional Repression of MFG-E8 Causes Disturbance in the Homeostasis of Cell Cycle Through DOCK/ZP4/STAT Signaling in Buffalo Mammary Epithelial Cells

**DOI:** 10.3389/fcell.2021.568660

**Published:** 2021-04-01

**Authors:** Arvind K. Verma, Syed A. Ali, Parul Singh, Sudarshan Kumar, Ashok K. Mohanty

**Affiliations:** Proteomics and Cell Biology Lab, Animal Biotechnology Center, ICAR-National Dairy Research Institute, Karnal, India

**Keywords:** milk fat globule epithelial growth factor-8, cell proliferation, quantitative proteomics, cytoscape, KEGG, oncogenesis

## Abstract

The mammary gland is a unique apocrine gland made up of a branching network of ducts that end in alveoli. It is an ideal system to study the molecular mechanisms associated with cell proliferation, differentiation, and oncogenesis. MFG-E8, also known as Lactadherin, is a vital glycoprotein related to the milk fat globule membrane and initially identified to get secreted in bovine milk. Our previous report suggests that a high level of MFG-E8 is indicative of high milk yield in dairy animals. Here, we showed that MFG-E8 controls the cell growth and morphology of epithelial cells through a network of regulatory transcription factors. To understand the comprehensive action, we downregulated its expression in MECs by MFG-E8 specific shRNA. We generated a knockdown proteome profile of differentially expressed proteins through a quantitative iTRAQ experiment on a high-resolution mass spectrometer (Q-TOF). The downregulation of MFG-E8 resulted in reduced phagocytosis and cell migration ability, whereas it also leads to more lifespan to knockdown vis-a-vis healthy cells, which is confirmed through BrdU, MTT, and Caspase 3/7. The bioinformatics analysis revealed that MFG-E8 knockdown perturbs a large number of intracellular signaling, eventually leading to cessation in cell growth. Based on the directed network analysis, we found that MFG-E8 is activated by CX3CL1, TP63, and CSF2 and leads to the activation of SOCS3 and CCL2 for the regulation of cell proliferation. We further proved that the depletion of MFG-E8 resulted in activated cytoskeletal remodeling by MFG-E8 knockdown, which results in the activation of three independent pathways ZP4/JAK-STAT5, DOCK1/STAT3, and PIP3/AKT/mTOR. Overall, this study suggests that MFG-E8 expression in mammary epithelial cells is an indication of intracellular deterioration in cell health. To date, to the best of our knowledge, this is the first study that explores the downstream targets of MFG-E8 involved in the regulation of mammary epithelial cell health.

## Introduction

Milk fat globule-EGF-factor is a 72-kDa secreted glycoprotein initially recognized in MFGM released into milk by MECs. It was identified to function as a bridging protein for apoptotic cells and phagocytes, leading to the subsequent engulfment and clearance of apoptotic cells. However, several other studies report that MFG-E8 is a multifunctional molecule shown to be released by a variety of cell types such as macrophages, immature dendrocytes, myoepithelial, endothelial, retinal, intestinal epithelial cells (Ceriani et al., 1983; Raymond et al., 2009; Zhou et al., 2018; Chopra et al., 2020). It was also found to be expressed at high levels in many tumor types (Carmon et al., 2002; Neutzner et al., 2007; Ko et al., 2020; Yamazaki et al., 2020). Hitherto, the most critical described function of MFG-E8 is to regulate immune homeostasis through the phagocytosis of apoptotic cells by signaling through α_*v*_β_3__–__5_ integrins linking phosphatidylserine at the surface of membrane vesicles (Oshima et al., 2002) of apoptotic cells (Hanayama et al., 2002; Lotfan et al., 2018; Peterman et al., 2019).

During the inflammatory process, it mediates phagocytosis induced regulatory T cell response (Jinushi et al., 2007) and Mfge8^–/–^ mice develop the spontaneous onset of lupus-like disease and glomerulonephritis (Hanayama et al., 2004). In addition to the immunological process, it also involves multiple regulatory functions, including in humans, SNP in MFG-E8 is associated with SLE (Hu et al., 2009). This protein shares structural domain homology with Del-1 (developmental endothelial locus 1), constituting a two-gene family of α_*v*_β_3_ integrin ligands (Hidai et al., 1998). Leading to the binding of MFG-E8 to α_*v*_β_3_/β_5_ on vascular endothelial cells promotes VEGF-driven neovascularization (Silvestre et al., 2005) and knocks down suppresses glioma progression (Wu et al., 2020). Therefore, it drives us to study the impact of MFG-E8 for other functions, including the role in the crucial cellular proliferation and homeostasis through phagocytosis (Li et al., 2019).

Importantly, numerous investigations have demonstrated that MFG-E8 plays an essential role in mammary gland development, essentially harmonizing post-lactational mammary organ remodeling (Stubbs et al., 1990; Lönnerdal, 2003; Atabai et al., 2005; Hanayama and Nagata, 2005). The removal of apoptotic cells is necessary for developmental stages and cleansing the mammary gland during lactation from unwanted pathogens. In turn, these studies highlighted an essential function of MFG-E8 in maintaining homeostasis of the mammary gland stages for lactation and involution function (Stubbs et al., 1990; Lönnerdal, 2003; Atabai et al., 2005; Hanayama and Nagata, 2005). It was also reported that MFG-E8 transcripts increase in the mammary gland during pregnancy to lactation, suggesting its typical role during the glandular developmental transition (Oshima et al., 1999). Lately, we also identified in our DiGE based proteomics dataset that MFG-E8 is up-regulated in MECs isolated from the high milk yielding cows (Janjanam et al., 2014). Despite the several reports emphasizing the association between this molecule and various cellular physiologies, the exact mechanism, its intracellular targets, and downstream signaling circuits in MECs are not known. The interpretation of MFG-E8 mediated regulatory control on homeostasis in healthy and diseased conditions remains poorly understood.

Therefore, the present study aims to understand the essential molecular shifts under MFG-E8 signaling. In this work, we observed the effect of MFG-E8 repression on cellular physiology using iTRAQ based high throughput proteomics technique and respective biological assays. We determined the linkage of MFG-E8 to maintain cell cycle and cellular proliferation through the ZP4/JAK-STAT5, DOCK1/STAT3, and PIP3/AKT/mTOR pathways.

## Materials and Methods

### Cell Culture and Stable Knockdown of MFG-E8 Expression in BuMEC Cells

The BoMac cell line was the generous gift by Prof. J. R Stabel. The cells were cultured in RPMI medium with 10% (FBS) (Cat. No. A4766801, Gibco BRL, United States). The BuMEC was previously established in the lab [ExPASy accession: Buffalo 2012 (CVCL_M445)] and used for the experimental purpose. Briefly, cells were cultured in DMEM/F12 media (Cat. No. 11330032, Gibco BRL, United States) supplemented with 8% (FBS) (Cat. No. A4766801, Gibco BRL, United States), 5 μg/ml bovine insulin (Cat. No. I-035, Sigma, United States), 1 μg/ml hydrocortisone (Cat. No. H0888, Sigma, United States), 10 ng/ml EGF (Cat. No. E4127, Sigma, United States), 100 U/ml penicillin, and 5 μg/ml streptomycin (Cat. No. P4333, Sigma, United States) were incubated with 5% CO_2_ at 37°C in a humidified environment. The confluent monolayer after 4–5 days were sub-cultured at split ratio of 1:3 by seeding in 1 × 10^6^ cells/flasks (25 cm^2^ flasks) by trypsinization (0.5% trypsin and 0.05% EDTA). The fresh medium was changed in ever two alternate days regularly.

The mfge8 gene shown in Supplementary Figure 1 was used for the preparation of shRNA targets. The designed three shRNA MFG-E8_1 (TCCCACAAGAAGAACATATTT), MFG-E8_2 (CGGTCAGGAGATAAGATATTT) and MFG-E8_3 (CAACAGCGGCCTGAAGATTAA) oligonucleotides were annealed in pLKO.1-puro-CMV-tGFP Vector backbone individually and used for the transfection. For making the stable knockdown BuMECs (3 × 10^5^) cell line, initially, cells were cultured in 24 well plate at 70% confluence, transfected with 3 μg of a non-target scramble and targeted shRNA pLKO.1-puro-CMV-tGFP Recombinant plasmid using lipofectamine 2000 (Cat. No. 11668027, Invitrogen Thermo Fisher Scientific, Waltham, MA, United States) using manufacturer protocol. After 24 h, cells were shifted to complete medium containing 50 μg/mL of puromycin (Cat. No. A1113803, Invitrogen Thermo Fisher Scientific, Waltham, MA, United States) as a selective agent. After 48 h of transfection, cells were trypsinized and seeded at low density in puromycin-containing media and allowed to grow with regular media changed every 48 h. Colonies of transfected cells were selected and further propagated in the selection medium by retrograde passaging using clonal dilution till a stably transfected KD BuMEC_MFG-E8 cell line (KD_MEC) was established. The expression of GFP protein was observed under fluorescent microscope (NikonTE2000, Japan). The effect of the knockdown was confirmed by analyzing the low-level expression of MFG-E8 at different passages by quantitative real-time PCR. The cell line stably transfected with MFG-E8 shRNA pLKO.1-puro-CMV-tGFP was designated as KD_MEC_MFG-E8 (KD_MEC) and the control transfected with scramble pLKO.1-puro-CMV-tGFP plasmids were named as Puro_MEC_MFG-E8 (WT_MEC).

### Sample Preparation for Proteomic Analysis

For the preparation of the proteomics sample, KD_MEC_MFG-E8 (KD_MEC) and Puro_MEC_MFG-E8 cells (WT_MEC) were cultured for 80% confluence, washed three times with PBS. The cells were harvested using 500 μL of lysis buffer [50 mM Tris-Cl (pH7.8), 0.3% SDS, 200 mM DTT, 1 mM PMSF, 1 mM EDTA] containing a protease inhibitor cocktail (Sigma-Aldrich) and vortexed. Subsequently, sonication was performed using the 10 cycles (pulse ON 5 s and OFF 7 s) with an interval of 120 s. The undisrupted cells were removed by centrifugation at 2500 × *g* for 15 min, and the supernatant was transferred to another tube. Proteins were precipitated by adding 10 volumes of cold acetone followed by overnight incubation at −20°C. The precipitated proteins were collected by centrifugation at 12,000 × *g* for 20 min. The protein concentration in the cell-free extract was determined using a 2D Quant kit (GE Healthcare, Life Sciences), according to manufacturer protocol.

### Protein Digestion, iTRAQ Labeling, and RP-HPLC Fractionation

The iTRAQ labeling was performed as per the manufacturer protocol (AB Sciex, Marsh Rd., Foster City, CA, United States). Briefly, the lyophilized protein sample (100 μg) from each group was resuspended in 20 μl of 2% sodium dodecyl sulfate (SDS) in 1 M triethylammonium bicarbonate (TEAB). The samples then reduced in 50 mM tris-(2-carboxyethyl) phosphine (TCEP) for 1 h at 56°C and cysteine-blocked with 200 mM iodoacetamide (IAA) at room temperature in the dark for 30 min. The proteins were digested using trypsin (Mass Spec Grade, Promega, Madison, WI, United States) at 1:80 (trypsin: protein ratio) for 16 h at 37°C. The prepared peptides were iTRAQ labeled in duplicate pairs with KD_MEC_MFG-E8 (KD1-114, and KD2-116) and Puro_MEC_MFG-E8 (WT1-115, and WT2-117) for 1 h at room temperature. The reaction was stopped by adding deionized water, and the labeled samples were pooled. The final combined sample was fractionated by RP- HPLC (Agilent 1100) using an analytical column [Grace Smart RP, C18 (150 × 4.6 mm), 5 μm]. The mobile phase A (10 mM TEAB) and B (10 mM TEAB in 90% ACN) was used, respectively, for the peptides separation using following linear gradient: 0 to 2% in 5 min, 2 to 60% in 60 min, 60 to100% in 25 min and then 100 to 2% in 2 min. The collected 120 fractions were concatenated into 30 fractions, again lyophilized and reconstituted in 0.1% formic acid in water and subjected to desalting using C18 zip tip (Millipore, United States). Eluted peptides were lyophilized and re-dissolved in 0.4% FA and immediately used for MS/MS spectra generation.

### Electrospray Ionization Tandem Mass Spectrometry LC-MS/MS Analysis

The reconstituted peptides were used for shotgun LFQ and iTRAQ proteomics experiments. The peptides were separated using nano-LC (Nano-Advance, Bruker, Germany) through a trap column (Bruker Magic C18 AQ, 0.1 × 20 mm, 3 μm, 200 Å) and a nano-analytical column (Bruker Magic C18 AQ, 0.1 × 150 mm, 3 μm, 200 Å) coupled with captive ion source (Bruker Captive Spray tip) spray in Maxis-HD qTOF (Bruker, Germany) mass spectrometer (MS) for identification with high mass accuracy and sensitivity. The elution was performed with a flow rate of 400 nL/min in a continuous gradient of 5–75% acetonitrile over 135 min. In the solvent system; Solvent A was 100% water in 0.1% formic acid, and solvent B was 100% acetonitrile in 0.1% formic acid. For acquisition, the data-dependent mode was used in mass spectrometer operated into automatically switch between MS and MS/MS acquisition. The precursor ion MS spectra scan range of 300–1400 (m/z) was used in the Q-TOF with resolution *R* = 75, 000. The six most abundant precursor ions were searched for detection of different masses in the acquisition method and selected for fragmentation using collision-induced dissociation (CID) with a fixed cycle time of 3 s along with 2 min of release for exclusion filter (otof processing software, Bruker Daltonics). The mass spectrometry proteomics data have been deposited to the ProteomeXchange Consortium via the PRIDE [1] partner repository with the dataset identifier PXD022445.

### Data Processing for iTRAQ Analysis

With the above mentioned same parameter, the iTRAQ analysis was performed in the Maxquant with Andromeda environment. Simultaneously, the data were also analyzed using the TPP pipeline. For TPP analysis, the obtained otof generated raw (.d) files were converted to mzML format using MSconvertGUI using the default parameters. The converted mzML files were searched using the Trans-Proteomic Pipeline version 5.1.0 released on 2017-11-03 to in-house combined UniProt (Cow), and *Bubalus bubalis* (Buffalo) database together with common contaminant sequences were provided for MS/MS spectra search. The database was also appended with an equal number of decoy sequences (reversed proteins sequences from the original database). Initially, for the analysis, the peptide assignments were performed using multiple search engines using X! Tandem (with the *k*-score plug-in) (MacLean et al., 2006), SpectraST, and Comet. The multiple search engines parameters were adapted from our previously published work (Suhail et al., 2019). For all the search engines, the search parameters included semi-digested LysC and trypsin with two allowed miss cleavage, static modifications carbamidomethyl (Cys), and iTRAQ reagents (N-terminus and Lys), and dynamic modification oxidation (Met), Gln-pyro Glu and Glu-pyro Glu remaining parameter were kept as default. The minimum peptide length parameter was set to seven amino acid residue. Further, PeptideProphet and ProteinProphet algorithms were used in the pipeline to compute the probabilities score for both individually searched peptides and the respective proteins. The accurate mass model in PeptideProphet was used for high confidence peptide identifications to boost the probability of peptides (Deutsch et al., 2010). Another protein validation step was executed using both Peptide Prophet and Protein Prophet scores, where the protein was authenticated if it contains minimum two top-ranked peptides with each peptide probability score above 95% (Supplementary Figure 1).

All the search engine results were merged and validated using another computational method, termed iProphet. This method takes as the input of PeptideProphet spectrum-level results from multiple LC-MS/MS runs and then computes a new probability at the level of a unique peptide sequence [or protein sequence (Nesvizhskii et al., 2003)]. This framework allows for the combination of results from multiple search tools. It takes into account other supporting factors, including the number of sibling experiments identifying the same peptide ions, the number of replicate ion identifications, sibling ions, and sibling modification states. A model of iProphet performance for the number of correct identifications *versus* error is shown in Supplementary Figure 1. An iProphet probability of more than 0.95 was used as the cutoff for the final identification of the protein. For protein quantitation, ≥2 unique peptides per protein were considered to ensure high-quality quantitation.

### Bioinformatics Analysis and Network Construction

The protein--protein interaction networks were constructed utilizing DEPs, to examine the complex interaction and prediction of pathways. We retrieved the whole protein sequences using an in-house made python script from the UniProt database for all proteins, and these proteins were mapped for protein--protein interactions networks using STRING v 10.0 [highest confidence (0.90)], Kyoto Encyclopedia of Genes and Genomes (KEGG)^1^, GeneMANIA, and Reactome. Further, Cytoscape 3.2.1 was used to visualize networks and were manually for biologically relevant processes. We used R package ggplot2 for creating the volcano plot while PCA plots were prepared using the factoextra package. Two separate PCA plots were created, first is the individual group component analysis for discrimination of Wild type cells and Knockdown cells (i.e., complete set of experiments). Second is the biplot of variables PCA, and it takes into account the consideration of all quantified proteins (i.e., complete set of proteins).

To further elucidate the biological inference of the enrichment terms and associated pathways, we employed Cytoscape plug-in ClueGO. It combines the pathways and gene ontology terms in functional networks, which represent the significance of the relationship between the pathways. The degree of nodes is designated by the number of edges it is connected for a network. It functions to illustrate the relationships between the Gene Ontology (GO) terms based on their overlapping genes. The term-term similarity matrix was calculated to establish the connection strength between the terms based on the highest significance. This study uses the following parameters kappa score of 0.4, GO tree interval 3 minimum level, 8 maximum levels, GO term/pathway selection four minimum genes, 3% genes. For statistical analysis, two-sided hypergeometric distribution tests were performed with Benjamini and Hochberg false discovery rate (FDR)-correction at <0.05 significance level.

The gene-term enrichment analysis was performed with Gene Ontology Enrichment Analysis Software Toolkit (GOEAST) released 2016-07-15 Version 11.1. The software calculated the fold enrichment based on the expected value. The expected value is the number of proteins expected in a particular GO category, based on the reference list available in the database. Subsequently, it calculates the fold enrichment based on the expected values for a particular GO term, and the probability is determined using a binomial statistic for *p*-values with a minimum cutoff of 0.05.

### Transcriptional Analysis at mRNA Level by qRT-PCR

The qRT-PCR analysis was performed, as explained earlier (Ali et al., 2017). Cells were grown at 80% confluence, three times cold PBS washed, mild trypsinized and pelleted by centrifugation at 400 × *g* for 2 min and samples were collected for RNA isolation. RNA was extracted using the RNAeasy mini kit (Qiagen, Germany) followed by cDNA synthesis using cDNA Synthesis kit (MBI, Fermentas). The reactions were carried out for 25 μl total volume containing 1 μl template, 10 nM of 1.25 μl primers, 12.5 μl master mix (Thermo Fisher Scientific, United States) and 9 μl nuclease-free water keeping the standard conditions in 35 PCR cycle (Ali et al., 2017). The associated fold change was measured using the 2–ΔΔCT method. The standard *t*-test statistic was applied for the comparison of the expression of the gene (*p* ≥ 0.05). Data were analyzed using MS Excel 2007 and Prism software 5.01 (GraphPad Software, United States).

### Cell Viability Tests: MTT; BrdU; Caspase Activity Assay for Apoptosis Determination

To assess the cell viability of MFG-E8 KD and WT cells, we used four different principles based on apoptosis determination assay. For all the assays, cells were initially seeded in a cell count of 1 × 10^5^ cells/ml in a 96-well plate with different intervals of time (12, 24, 36, 48, 60, and 72 h) including respective controls containing complete medium without cells. For MTT-assay [3-(4,5-dimethylthiazol-2-yl)-2,5-diphenyltetrazolium bromide (MTT, Sigma)] MTT solution of 0.5 mg/ml dissolved in PBS was added in accordance to the mentioned time intervals and incubated for 2 h at 37°C in 5% CO_2_ followed by addition of the DMSO to dissolve the precipitated formazan and absorbances were measured at 570 nm using NanoQuant plate reader (TECAN, Infinite 200 PRO, Mannedorf, Switzerland).

The BrdU (5-bromo-2′-deoxyuridine) assay was performed according to the manufacturer’s protocol using the assay kit (Cat No. Q11A58, Calbiochem, United States). As mentioned above, cells were seeded in a 96-well plate but with 100 μl medium containing the density of 200 cells per well. The BrdU labels were incubated for 2 h in the growing cells (KD and WT transfected) for integration, followed by measuring the absorbance at 540 nm using the Nano-quant plate reader.

To measure the degree active Caspase- 3/7, we used Caspase-Glo 3/7 activity assay (Promega Cat No. G8091, Madison, WI, United States) according to the manufacturer’s protocol with minor changes as also described previously (Kaur et al., 2017). Briefly, cells were seeded and incubated as mentioned above in 96-well plate, and after completion of the individual incubation period, 100 μL of Caspase-Glo 3/7 reagent was added. Further, the plate was incubated for 3 h at room temperature, and luminescence intensities were measured using excitation wavelength 492 nm and emission wavelength 535 nm.

### Cell Confluence, Wound Healing, and Transwell Assay

The number of 4 × 10^4^ KD, WT, KD + HA 14-1, and WT + HA 14-1 BuMEC cells were cultured in 24 well cell culture plates to ∼10% of confluence. Cell proliferation was monitored every 7 h using Bio-Rad TC20 automated cell counting system (Bio-Rad, CA, United States) up to 56 h for over 9-time points.

The migratory behavior of KD and WT BuMEC cells was evaluated using the wound healing scratch assay. An equal number of cells were seeded on 6-well plates in six biological replicates and allowed to reach 90% confluency. Upon reaching the suitable confluent stage the cells were then 12 h serum-starved followed by creating the wound by spanning the line at the bottom of the individual wells by using a sterile p-20 pipette tip. All the wells were rinsed gently with DPBS and then one set of the KD and WT cells were treated with HA 14-1, a well-known apoptosis inducer. As mock control cells were also preincubated with DMSO alone. The quantification was manually completed in the scratch cross-sectional area by measuring the number of cells migration.

In the transwell assay, using transwell buckets (Millipore, United States) was covered to the bottom of the inner wells. A cell suspension (5 × 10^4^ cells in 200 μL of DMEM without FBS) was added to the upper chamber, and DMEM with 10% FBS (500 μL) was added to the lower chamber. After 15 h, the migrated cells were stained with hematoxylin. The stained cells were analyzed by microscope in five randomly selected fields/assay. The Image J software counted the cell numbers. Values represent the means ± SD from six replicates.

### *In vitro* Phagocytic Assay

The phagocytosis assay was performed using BoMAC and the BuMEC cell line (KD and WT) with the help of a previously published protocol (Hanayama et al., 2002). The apoptosis was induced by HA 14-1 in the assay. The respective BuMEC (1 × 10^6^ Cells) KD or WT with or without apoptotic inducer were added to 2 × 10^4^ macrophage cells previously cultured in eight well Lab-tec II chamber slides and incubated for 1.5 h followed by the fixation using 1% paraformaldehyde and TUNNEL staining was performed. The TUNNEL positive apoptotic cells per phagocytes were counted (phagocytosis index) by light microscopy in a total of 200 phagocytes, and the relative index was determined.

### Statistical Analysis

These statistical calculations were performed using the GraphPad Prism 4 software package (GraphPad Software, Inc., La Jolla, CA, United States). Data were shown in the form of bar plot as mean with standard error of mean (±SEM) with relative 100 percent to control. Statistical significance for MTT, BrdU, Caspase 3/7, and cell grown confluence assay was determined using a two-way ANOVA test with Bonferroni post-test correction for FDR-correction at <0.05 significance level indicated by asterisk marks. For wound healing, transwell, and phagocytosis assay one-way ANOVA test with repeated measures along with post Turkey test for FDR-correction at <0.05 significance level indicated by asterisk marks.

For mass spectrometer and qRT-PCR results, data are represented as relative log_2_ fold change. Transcripts CT values were normalized against the housekeeping gene β-actin, and fold change was calculated using 2^–Δ^ ^Δ^ CT method. All experiments were performed in triplicates. The significance of the differences in expression levels was tested using the non-parametric Mann–Whitney *U* test ^∗^*p* < 0.05, ^∗∗^*p* < 0.01, ^∗∗∗^*p* < 0.001.

## Results

### Analysis of MFG-E8 Protein Sequence and Evolutionary Relationship

**FIGURE 1:**
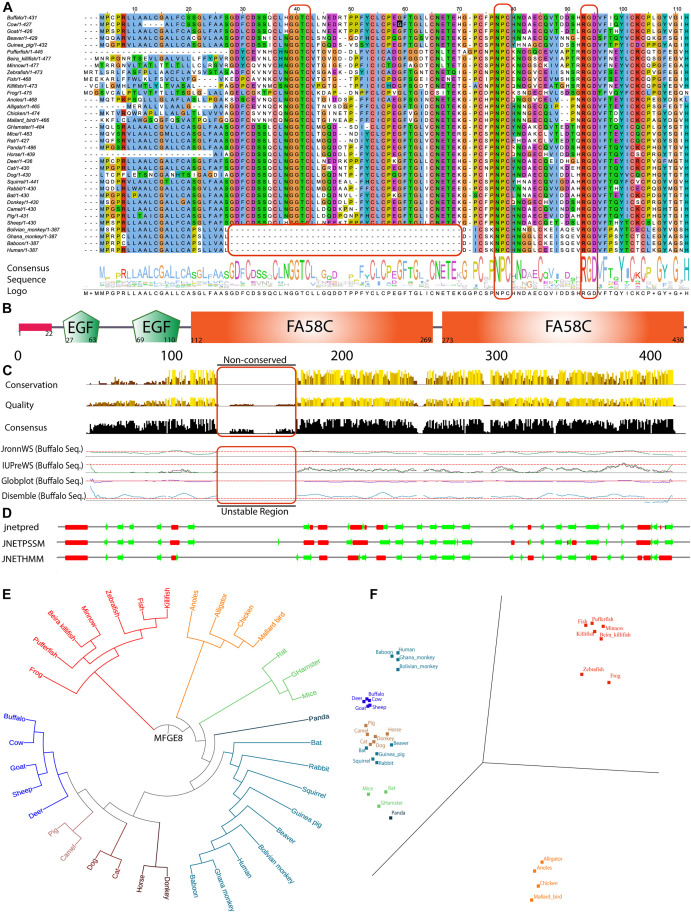
*In silico* based MFGE8 protein sequence characterization. **(A)** Multiple sequence alignment (MSA) of 35 organisms used from UniProt non-redundant and reviewed database. Total numbers of amino acid residue present in the individual sequence were reported in front of the organism name, and MSA is clustal color-coded. The consensus sequence logo was shown in the downside of the alignment, and the conserved nature was depicted using the size of the amino acid single alphabet symbol. **(B)** The structure of the MFGE8 protein containing the complete common domains observed in all programs. **(C)** The full-length sequence quality, conservation, and consensus nature of MSA are shown in comparison with 35 organism-specific MFGE8 protein. The buffalo specific MFGE8 protein sequence disordered behavior was analyzed using four algorithms (JronnWS, IUPreWS, Globplot, and Disemble), keeping the parameter defaults for all of them. The graph showed consistent results among the programs. **(D)** The secondary structure was predicted for a full-length MFGE8 sequence with the help of three independent programs (jnetpred, JNETPSSM, and JNETHMM). The red mark shows the α-helical structures, while the green arrows show the β-sheets structures in the MFGE8 protein. **(E)** Phylogenetic tree analysis of all 35 organisms used using distance tree. **(F)** Principal component analysis of MSA for all 35 organisms using BLOSUM 62 algorithm.

The mammary gland remodeling is a highly programmed, genetically controlled process involving cell death during involution that is both spectacular in scope and well-organized in execution. As shown in multiple reports that MFG-E8 is an essential protein for the successful accomplishment of the involution process (Stubbs et al., 1990; Lönnerdal, 2003; Atabai et al., 2005; Hanayama and Nagata, 2005). Our goal of this study is to determine the associated regulatory proteins and signaling pathways linked with MFG-E8 protein through its knockdown in buffalo MECs. As the information on the role of MFG-E-8 in bovine is scanty, we examined the sequence and its domain organization for evolutionary conservation (accession number NP_001277850.1). The same mfge8 full-length gene sequence used in multiple sequence alignment (MSA) was used for the preparation of shRNA for knockdown experiments (Supplementary Figure 1). The MSA of MFG-E8 from 35 organisms showed the absolute conservation of the RGD motif that is involved in cellular interactions with cell-surface integrins (Taylor et al., 1997), but we argued that its presence may be fortuitous and may imply for another cellular process. Likewise, we determined another NPC novel conserved motif among the organisms, not discussed in the previous literature. Nonetheless, the deletion of 42 amino acid residues was observed (28th to 70th aa) in the MFG-E8 sequence of a human, baboon, Ghana monkey, and Bolivian monkey, suggesting its role in evolutionary selection. Interestingly, in the same region in remaining all organisms, a conserved GGTC sequence was observed. The result implies that the later got diverged during evolution and lost 42 aa length peptide (Figure 1A).

Further using full-length protein MSA (Supplementary Figure 1) and different databases, we determined the five conserved domains namely: 1 signal peptide, 2 EGF domains (respective e-values are 0.000197 and 0.00000435), and 2 FA58C domains (respective e-values are 6.56e-41 and 2.01e-36) in the protein sequence which were found consistent among all 12 databases and algorithm searched (Figure 1B). Rest all other identified domains have been detailed in the Supplementary Table 2a. The conservation, quality, and consensus among protein sequences revealed that the residues from 115th to the 185th amino acid at the N-terminal is non-conserved. The results were consistent, which were validated by the four independent algorithms for the calculation of protein disorderliness (Figure 1C). The prediction of the secondary structure identified eight α-helix and twenty-two β-sheets, and these results were consistent among three different secondary structure programs (jnetpred, JNETPSSM, and JNETHMM) (Figure 1D). Further, the determination of motif recognition analysis using the motif-x algorithm showed the total identification of eighteen motifs corresponding to eight specifics for phosphorylation and ten for glycosylation (for detailed motifs information, see Supplementary Figure 2). The phylogenetic analysis of MFG-E8 from 35 organisms showed that buffalo is highly close to bovine, goat, sheep, and deer and comes in one cluster out of eight classified groups. The results were validated with the help of principal component analysis (PCA) analysis using the BLOSUM62 model of the MSA. In the PCA plot, a similar sequence lies close to each other. We identified the highly divergent nature of MFG-E8 in alligator, anoles, chicken, and mallard birds, which were grouped into a separate cluster (Figures 1E,F). Altogether, these results suggest that intradomain regions are conserved among the organisms, but many differences were observed in the interdomain areas.

### Impact of MFG-E8 Repression on the Morphology and Phagocytic Activity of MEC

**FIGURE 2:**
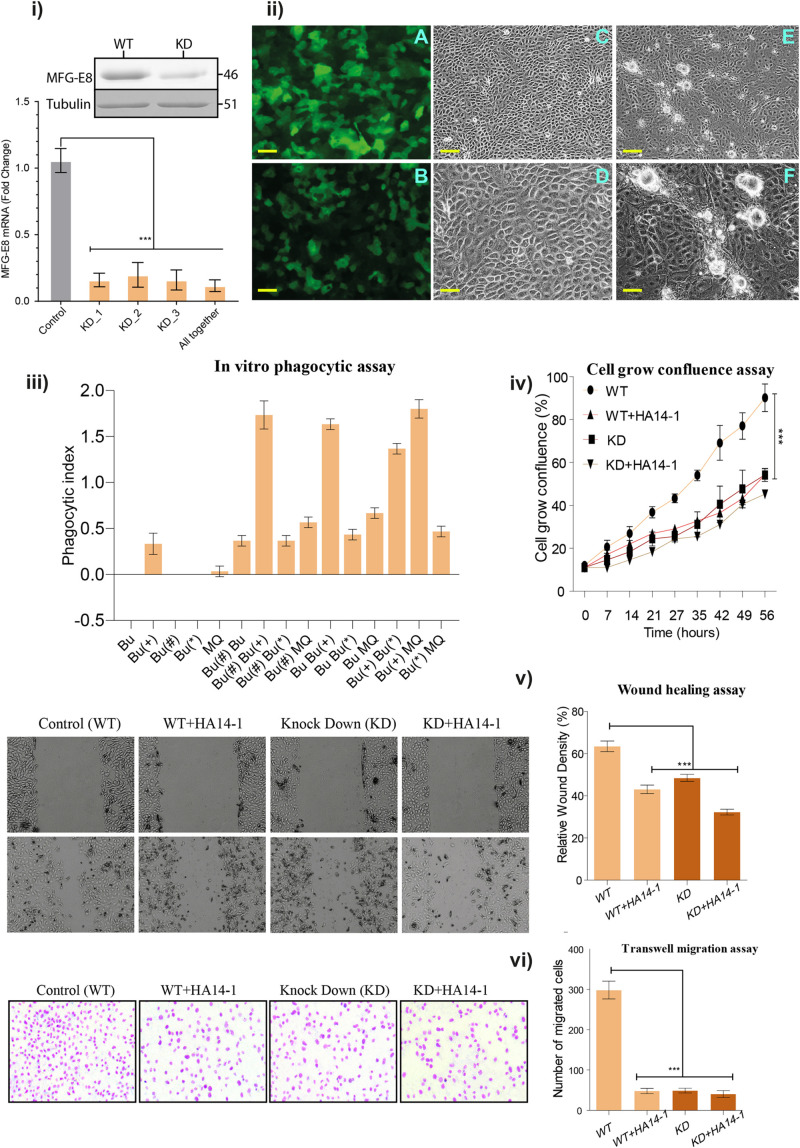
**(i)** Quantitative real-time PCR assay: Real-time PCR reaction was performed using three independent shRNA (KD_1, KD_2, and KD_3) and in a combination of all together. All the reactions were performed in three biological replicates and repeated at three replicates, and values are shown in ± SEM as error bars. **(ii)** Stable transfection of buffalo mammary epithelial cells (BuMECs) cells. **(A)** Transfected BuMEC cells over-expressing non-target scramble shRNA pLKO.1-puro-CMV-tGFP vector. **(B)** Transfected BuMEC cells with MFG-E8 specific shRNA containing pLKO.1-puro-CMV-tGFP vector, respectively. **(C)** Transfected BuMEC cells over-expressing non-target scramble shRNA pLKO.1-puro-CMV-tGFP vector at 72 h of growth, magnification 200×. **(D)** The same panel-C cells at higher magnification 600×. **(E)** Transfected BuMEC cells with MFG-E8 specific shRNA containing pLKO.1-puro-CMV-tGFP vector at 72 h of growth, magnification 200×. **(F)** The same panel-E cells at higher magnification 600×. Scale bar is 100 μm. **(iii)** *In vitro* phagocytic assay. The co-culture phagocytic assay was performed to determine the phagocytosis ability in the absence of MFG-E8 and the induction of apoptotic inducer (HA 14-1). The graph axis is denoted with Bu (normal or wild type BuMEC cells), Bu(+) (apoptotic induced BuMEC), Bu(#) (scrambled shRNA-MFG-E8 BuMEC cells), Bu(*) (MFG-E8 knockdown BuMEC cells), and MQ (BOMAC). **(iv)** Cell grown confluence assay. The WT and KD cells were used along within the presence or absence of HA14-1 inhibitors. The cells were grown with 7 h time interval for 8-time points up to 56 h. The experiments were performed in three independent biological replicates. **(v)** Wound healing assay. The same four conditions were used for the wound healing assay. All the reactions were performed in three biological replicates and repeated at three replicates, and values are shown in ± SEM as error bars. **(vi)** Transwell migration assay. The migration behavior of BuMEC upon knockdown and normal condition along with and without HA 14-1 inhibitor after 48 h was detected by wound healing assay. Cell migration was calculated as described in Section “Materials and Methods.” Values are normalized to their respective 0 h time point and presented as means ± SEM of three independent experiments. All the reactions were performed in three biological replicates and repeated at three replicates. Values with ***p < 0.001 are statistically significant. ns, non-significant.

Phosphatidylserine-MFG-E8 axis mediated cell death modality is evolutionarily conserved, which has been recently explained (Birge et al., 2016). In support, we identified the conserved MFG-E8 domains in all the species, signifying for the necessary cellular proliferation process and involvement in phagocytosis. The transfection, retrograde passaging, and continuous selection using puromycin antibiotic resulted in stable expression of shRNA. The presence of GFP in the vector helped for the positive selection of transfected clones. The qRT-PCR analysis showed 90-fold down-regulation in the expression of MFG-E8 transcripts (Figure 2i) and the same results were validated by western blot analysis. The dense expression of GFP shows the expression of a scramble as well as MFG-E8 specific shRNAs (Figures 2ii A,B).

Interestingly, the low expression level of inherent MFG-E8 protein resulted in the formation of globule-shaped structures that are not seen in the healthy or scrambled shRNA transfected cells (Figures 2ii C,D). However, the formation of these structures was prominent at 72 h in KD BuMEC cells (Figures 2ii E,F). To exclude the possibility that GFP is stimulating these globules aggregation, we transfected non-target scramble shRNA GFP vector to BuMEC cells as a negative control, and no such structures were observed (Figures 2ii C,D). To understand and discover the MFG-E8 associated interconnection of pathways in the regulation of MEC proliferation, apoptosis, and transformation, we performed iTRAQ based comprehensive quantitative proteomic analysis between KD_MEC and WT_MEC and validated the findings through multiple *in vitro* assays as discussed later.

As MFG-E8 was previously described as a bridging molecule between macrophages (MQ) and apoptotic cells (Oshima et al., 2002), we determined the phagocytic index of MFG-E8 knocked down MEC cells. For the control, we performed the assay alone for Bu (Normal or wild type BuMEC cells), Bu(+) (Apoptotic induced BuMEC), Bu(#) (scrambled shRNA-MFG-E8 BuMEC cells), Bu(^∗^) (MFG-E8 Knockdown BuMEC cells), and MQ (BOMAC) cell. Surprisingly, apoptotic induced BuMEC cells showed the phagocytosis behavior but not others. For assay we tested two separate control one with scramble shRNA and other with WT cells. The results showed the minimal phagocytic index in Bu Bu(^∗^) and Bu Bu(#) (0.42) than Bu(^∗^) MQ (0.49) and lastly to Bu(#) MQ and Bu MQ (0.73) (Figure 2iii). The suggested reasons for low phagocytosis index in MFG-E8 knocked down Bu(^∗^) cells may be the low expression of MFG-E8 or the decreased level of its associated receptors. Later the same results got confirmed in the proteomics data.

On the other hand, the maximal phagocytosis index was seen in the apoptosis induced Bu(+) MQ (1.81), which was used as a positive control. Unexpectedly, we observed the higher index in apoptosis caused Bu(+) Bu(^∗^) and Bu(#) Bu(^∗^), co-cultured (1.50) and in apoptotic induced Bu Bu(+) and Bu(#) Bu(+) (1.21). The detailed information about the ANOVA results has been provided in the Supplementary Table 2b. The phagocytosis assay results support previous findings that the healthy epithelial cells act as amateur phagocytes and are involved in the clearance of apoptotic cells through the expression of MFG-E8 (Monks et al., 2005, 2008).

Further, we examined the cell growth percentage in KD and WT cells up to 56 h; the results confirmed the significant high growth disparity in WT cells *p* < 0.001 at 56 h in relation to MFG-E8 KD. Subsequently, we tested the growth rate in the presence of the apoptotic inducer HA14-1, it resulted in the reduction of the KD cell population at 56 h to 52% growth, and WT treated with HA14-1 at 56 h to 55% growth compared to control with significant difference *p*-value <0.001. In comparison to wild type, the initial significant difference for all three conditions was observed from 21 h of the cell growth assay (Figure 2iv). Next, we performed the wound healing assay using the same four conditions. The analysis of variance showed that in the first comparison of WT versus WT + HA14-1 mean difference-20.33 (*P* < value 0.0001), in second comparison WT versus KD, mean difference-15.00 (*P* < value 0.0001), and in the third comparison WT versus KD + HA14-1 mean difference-31.17 (*P* < value 0.0001) (Figure 2v). The results describe that KD or treatment with the HA 14-1 inhibitor showed a remarkable reduction in the proliferation ability during would healing assay. Comparable results were perceived in the transwell migration assay with a significant difference with WT cells for cellular migration (Figure 2vi).

### Widespread Proteome Changes Triggered by Ablation of MFG-E8

**FIGURE 3:**
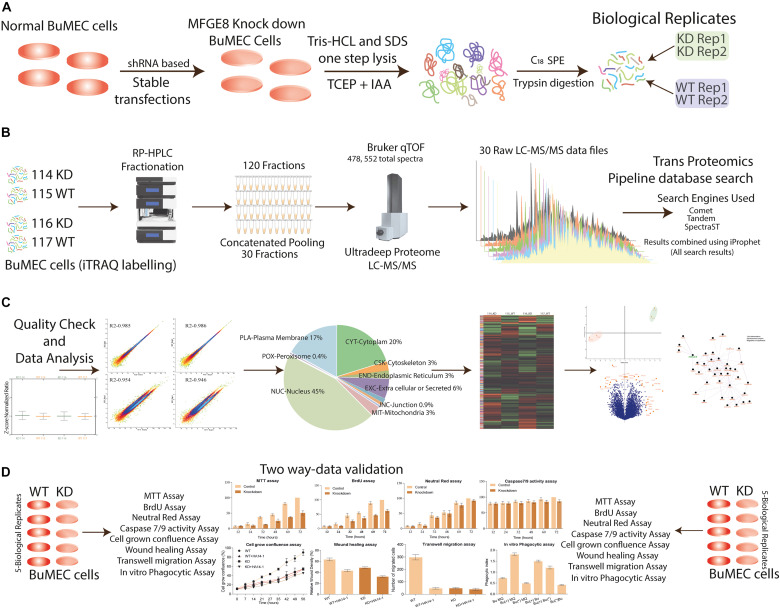
Experimental design and quantitative proteomics workflow allow a comprehensive analysis of MFGE8 affected proteome. **(A)** BuMEC cells were transfected with shRNA for stable knockdown of MFGE8 protein expression, stably transfected cells grown for 120 h followed by quick lysis, protein extraction, quantification, and trypsin digestion for peptides generation in two biological duplicates (see the section “Materials and Methods”). **(B)** Peptides were labeled with iTRAQ labels, which are 114, 116 for KD (knockdown cells), and 115, 117 for WT (wild type cells treated with scrambled shRNA) followed by reversed-phase UHPLC based peptide fractionation. The fractions were concatenated pooled into 30 final fractions, and iTRAQ labeled peptides were analyzed by high-resolution qTOF-LC-MS/MS. The database search analysis was performed by using three search engines (Comet, Tandem, and SpectraST) in the environment of the transproteomics pipeline, further, the description of raw data processing as described in Section “Materials and Methods”. **(C)** Outline of mass spectrometer data processing and analysis is shown; necessary detailed methodologies are provided in the Section “Materials and Methods.” For relative quantification, data were filtered and normalized to make sure fair evaluation across all duplicate biological samples. The resulting identified proteins were used for their localization in the cell compartments. The heat map was generated for clustering the group of proteins. Principal component analysis was performed to verify the isolation of the components of different labels. Finally, the volcano plot was graphed to identify the significantly differentially express proteins. These DEPs were used for network analysis, and lastly, the biological MFGE8 based regulatory pathway was predicted. **(D)** The validation using the different biological assays.

We employed the loss of function strategy and optimized the iTRAQ based global quantitative proteomics workflow to unravel the regulators of MFG-E8 mediated signaling pathways crosstalk. The protein samples were prepared from 5 days grown MFG-E8 deficient and wild type epithelial cells, followed by digestion, iTRAQ labeling, fractionation, and LC-MS/MS-based identification and quantitation. The combined workflow trans proteomics pipeline analysis enabled the identification of 4,78,552 total spectra using three search engines, which represented 12,649 proteins having an iProphet probability of ≥0.999 and error rate <0.00001 (Figures 3A,B). The Libra based relative quantification of non-redundant protein entries resulted in 8858 DEPs (*p*-value < 0.01). Only those proteins were selected for analysis, which qualified the inclusion criteria of ≥2 peptides, minimum cutoff MS/MS count 2, minimum of seven amino acid residues, and iProphet probability ≥ 0.95 per peptide (Figure 3C). Subsequently, the precision in the data acquisition was determined using statistical correlation among technical and biological replicates, which have been shown as multiple densities scatter plots (Supplementary Figure 3). The Pearson correlation *R*^2^ value of 0.985 and 0.986 was observed between the biological replicates of KD and WT cells, respectively (Supplementary Figure 4). The plots show the precision in acquired MS/MS spectral intensities used for the quantification. A list of identified proteins, iProphet protein probability, sequence coverage, peptide sequence, Libra quantification values, and fold change values between MFG-E8_KD and Wild type control cells has been provided in Supplementary Table 1. Proteomics findings were verified with results obtained from various cellular assays (Figure 3D).

**FIGURE 4:**
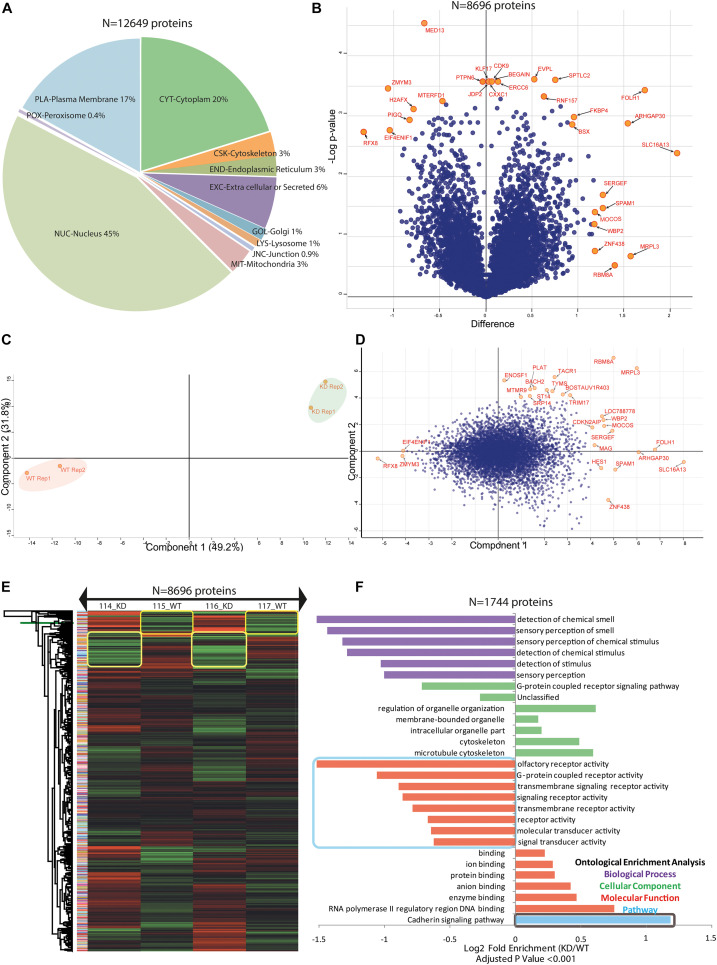
BuMEC in-depth proteome analysis. **(A)** A pie chart shows the different percentage of occupancy of the identified proteome in compartmental based localization of epithelial cells. See also Supplementary Table 5. **(B)** Volcano plot shows the significantly differentially expressed proteins and extremely affected proteins were labeled with the orange color circle with named in red color. The two-sided *t*-test was calculated using artificial within-group variance S0 equals zero. Further truncation was performed using a permutation based FDR equals 0.01, and the number of randomizations equals 250. The *X*-axis is the *t*-test difference values while the *Y*-axis is –log of adjusted *p*-values. For detailed data, see also Supplementary Table 1. **(C)** Principal component analysis shows the proteome data isolation of the components of different iTRAQ labels for biological duplicates samples. Highlighted color reflects the replicate groups; the red color is for wild type cells (WT), and green color is for knockdown cells (KD). **(D)** The biplot of PCA shows proteome data for comparison of component 1 (*X*-axis) to component 2 (*Y*-axis) for WT and KD cells in differentially identified proteins. Blue dots that are closely associated are highly correlated in terms of the observed proteome, and extremely isolated orange circles are separated proteins from PC1 and PC2 and were labeled as red color. **(E)** Heat map presentation of a hierarchical cluster of all 8696 proteins that show significantly different (*p* ≤ 0.05) relative abundances in WT versus KD cells. The green color depicts low while red color depicts high expression levels. **(F)** Protein ontologies enrichment analysis is shown in the form of a bar graph. Biological process (BP), cellular component (CC), molecular functions (MF), and pathways were color-coded with bars. All the enriched values were log_2_ transformed, and the adjusted *p*-values used as <0.001. For all groups, the overall number of fold enrichment is shown in comparison to the reference database, and detailed information is provided in Supplementary Table 1.

The profiled ultra-deep MEC proteome was cataloged, and individual proteins were predicted for its localization in the cellular compartments based on the consensus motifs available in the full-length protein sequences. Remarkably, we identified 45% of the whole proteome to be associated with the nucleus, while 20% of proteins were from the cytoplasm. With the help of our optimized proteomics workflow, including the microwave energy pretreatment of the proteins before digestion for optimal cell lysis, it allowed us to identify 17% of plasma membrane protein without any additional enrichment step. The remaining 18% were distributed in the other cellular compartment including peroxisome 0.4%, cytoskeleton 3%, endoplasmic reticulum 3%, Golgi 1%, lysosome 1%, junction proteins 0.9%, mitochondrial 3%, and extracellular or secreted proteins 6% (Figure 4A). The downstream statistical analysis in the R- environment using a two-sample *t*-test based modified SAM-test showed significant proteins in the volcano plot (Figure 4B). For all DE proteins corrected *p*-values were calculated assuming the equal variance in biological replicates while keeping the *fudge factor* s0 = 1 and FDR = 0.01, as the constant parameters to locate out the “*t*-test Significance,” “−Log *t*-test *p*-value,” “*t*-test Difference” and “test statistic” for all entries. The volcano plot provided an estimate for both sided significance values of proteins (Supplementary Table 1). Our statistically defined data portrayed that the knockdown of MFG-E8 changes the dynamics of proteome expression in MECs, indicating its crucial role in basic cell proliferation.

The majority of highly down-regulated proteins in KD_MEC cells included RFX8, SIX6, ELP3, ZMYM3, EIF4, ENIF1, MYH14, DNAH11, TSBP, CBLN4, CNP, OR14C36, and CD2BP. They belong to protein classes of cytoskeletal protein (PC00085), transferase (PC00220), and transcription factor (PC00218), hydrolase (PC00121), an enzyme modulator (PC00095) (Supplementary Table 3). The up-regulated proteins in KD_MEC cells included LYVE1, FOLH1, PRKCSH, TRPV3, RING1, DOCK1, PLTP, SLC16A13, LENG1, and DMTN which are involved in the functions of transporter activity (GO:0005215), receptor activity (GO:0004872), signal transducer activity (GO:0004871), catalytic activity (GO:0003824) (Supplementary Table 4). The proteins perturbed by the suppression of MFG-E8 expression in the epithelial cell showed the significant rearrangement of 572 membrane trafficking, 220 ion channel-specific, and 202 cytoskeleton proteins in KEGG annotation (Supplementary Figure 5 and Supplementary Table 5). The variety of ion channels include calcium-activated potassium channel, potassium voltage-gated channel, potassium channel regulatory protein, epithelial chloride channel, sodium channel protein, amiloride-sensitive sodium channel, transmembrane channel-like protein, short transient receptor potential channel 1, transient receptor potential cation channel, voltage-dependent anion-selective channel, and ATP-sensitive inward rectifier potassium channel (Supplementary Table 1). Another interesting observation is the up-regulation of the dedicator of cytokinesis protein isoforms; DOCK1, DOCK2, DOCK3, DOCK4, DOCK6, DOCK8, DOCK9, DOCK10, and DOCK11. The cytoskeletal changes and dramatic upregulation of transporter activity proteins together probably contribute to the distortion in the shape of KD_MEC cells on the fifth day of the culture, as seen in Figure 2ii. The size of the MFG-E8 knocked down cells as measured in terms of area and perimeter over the different points of observation, i.e., 12, 72, and 120 h of cell growth showed significant expansion (*p*-value ≤ 0.001). It may be associated with ionic imbalance inside MECs due to perturbation in many membrane channel proteins.

Our previous results of LIF overexpression also showed the activation of these identified membrane channels proteins, which resulted in the formation of globule structures in cells (Figure 2ii and Supplementary Table 1) (Kaur et al., 2017; Ali et al., 2018). We assessed all DEPs using the PCA plot to gain insight for its distribution in component 1 (49.2%) and component 2 (31.8%) with the identification of orthogonally transformed of essential proteins, indicated in the red label (Figures 4C,D). The majority of the proteins identified as PCA outliers showed their involvement in the cell cycle regulation in consonance with GO analysis and KEGG annotation (Supplementary Tables 3, 4, 5, respectively). The comparison using hierarchical cluster analysis with Euclidean distance and average linkage with K-mean 300 clusters revealed significant changes in the expression level of all 8858 proteins (Figure 4E) which is same as shown in the volcano plot (Figure 4B and Supplementary Table 1; 4,025 up-regulated genes, 4,833 down-regulated genes, false-discovery rate-adjusted *P* < 0.05). Thus, our result strongly suggests that knockdown of MFG-E8 protein directs widespread cellular response that is reflected by the changes in the whole proteome profile of MEC, requiring additional investigation into prominent cellular pathways and linked proteins that can distinguish the assault control mechanism from the direct downstream effects of MFG-E8 knockdown.

We observed that the suppression of MFG-E8 expression increased the duration of the G0-G1 phase transition by 32.62% in comparison to WT_MEC 18.4%. However, the S-phase duration is shortened in KD_MEC from 65.85% to 47.20% (Supplementary Figure 6). To explain this effect, we looked at the Gene Ontology classification of DEPs by GO analysis (see Materials and Methods section). The investigation revealed significant enrichment of 530 GO terms with adjusted *P*-value < 0.05, including BP (321), CC (94), MF (86), Reactome/pathways (11), and protein class (18) terms, respectively (Supplementary Table 2). We further scrutinized the main GO terms simultaneously using fold change cut off (2.0-fold up and 0.5-fold down-regulated) and highly significant *P*-value < 0.01 in each category to visualize the pervasive impact of low MFG-E8 level in cells (Figure 4F). Surprisingly, we found the over-enrichment of the process for the negative regulation of intracellular signal transduction (GO: 1902532) and negative control of the cell cycle (GO: 0045786). It supports our observation of an increase in the cell cycle, doubling the time of KD_MEC cells (Supplementary Figure 6). It is important to note here that the over-representation of key GO terms significantly enriched in the biological process showed posttranscriptional gene silencing (GO: 0016441) and gene silencing by RNA (GO: 0031047) which proves the shRNA mediated MFG-E8 silencing in our experiment. Further exploration of results showed that MFG-E8 knocked down disturbs the regulation of Ras protein signal transduction (GO: 0046578), cell-substrate adhesion (GO: 0031589), extracellular matrix organization (GO: 0030198), DNA repair (GO: 0006281), protein autophosphorylation (GO: 0046777). The under-representation analysis indicated the predominant effect on nucleosome assembly (GO: 0006334), G-protein coupled receptor signaling pathway (GO: 0007186), natural killer cell activation involved in immune response (GO: 0002323), detection of stimulus (GO: 0051606), Fc gamma R-mediated phagocytosis (bta04666) providing the evidence for a low phagocytic index of KD cells.

**FIGURE 5:**
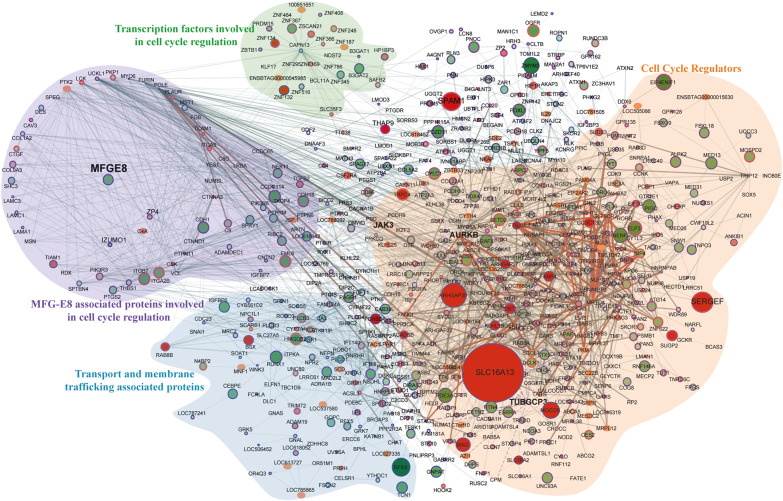
Protein-protein interaction map for top significantly (*p*-value < 0.001) expressed proteins. The total number of nodes is 682 with 2140 interacting edges, node color and size both show the up/downregulation, specifically, darker color and bigger size node defines the high value of the fold change. The smaller size node defines no observed fold change in proteomics data. The border of the nodes shows the significance of the lesser *p*-values < 0.001; thicker the border lowest is the assigned *p*-values. The thickness of the edges describes the experimentally identified co-expressed proteins in the database.

We mapped DE proteome (*P* < 0.05) using genemania, reactome, and string database in a single platform on Cytoscape into different cellular processes to illustrate interdependent proteins regulation under the signaling of MFG-E8 (Figure 5). Overall, with these results, we understand that the repression of the MFG-E8 affects a wide variety of proteins in MEC leading to a severe breach in cellular homeostasis.

### MFG-E8 Associated Proteome Regulates MEC Homeostasis Through Cytoskeleton Rearrangement

**FIGURE 6:**
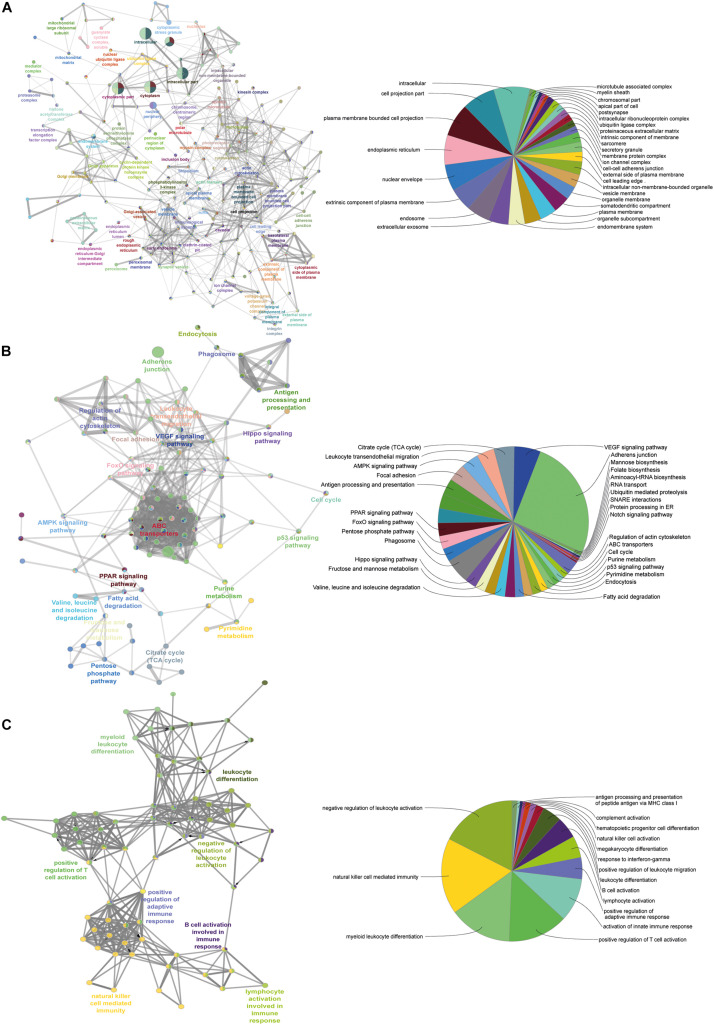
Pathway–pathway interaction analysis. A functionally grouped network of enriched categories was generated for the target genes. GO terms are represented as nodes, and the node size represents the term enrichment significance. Functionally related groups partially overlap. The node pie charts represent the molecular function, immune system processes, reactome analysis of targets. Only the most important term in the group was labeled. **(A)** Representative molecular function interactions among targets. **(B)** Representative reactome analysis interactions among predicted targets. **(C)** Representative immune system processes interactions among targets.

To understand the process of homeostasis in the absence of MFG-E8, we executed four powerful databases; Gene Ontology Enrichment Analysis Software Toolkit (GOEAST), ClueGO, DAVID, and KEEG (Figure 6). The over-enrichment of the prominent cellular component containing terms included GO:0005737 cytoplasms (12.35), GO:0016020 membrane (10.86), GO:0005654 nucleoplasm (9.06), GO:0005871 kinesin complex (7.77), GO:0005622 intracellular (6.72), GO:0005813 centrosomes (6.51), GO:0005925 focal adhesion (5.64), GO:0005829 cytosols (5.44), and GO:0008270 Ion channels (6.21). The Supplementary Tables 2, 3 enlist the details of all other enrichment. This analysis highlighted the negative regulation of cytoskeleton organization (GO:0051494), microtubule cytoskeleton organization (GO:0000226), which was in parallel to the finding of morphological changes in KD_MEC cells (Figure 2ii). The globular structure formation as a result of cytoskeletal perturbances has been reported in other studies as well, where stat3 mediated Na-K + channels are the key players (Ali et al., 2017). To gain insight on various pathways and their interconnections, we identified (Supplementary Figure 5) bta04510:Focal adhesion (10.19), bta04512:ECM-receptor interaction (10.18), bta04810:Regulation of actin cytoskeleton (10.18), bta02010:ABC transporters (5.90), bta04110:Cell cycle (5.18), bta04390:Hippo signaling pathway (4.67), bta00562:Inositol phosphate metabolism (4.31), bta04070:Phosphatidylinositol signaling system (4.15), bta04010:MAPK signaling pathway (3.97), bta04150:mTOR signaling pathway (3.72). The down-regulation of MFG-E8 thus proves that it is a central molecule in the maintenance of cellular homeostasis. A large number of cellular processes are interconnected through MFG-E8, among which structural components involved in focal adhesion, ECM receptor interaction, and actin cytoskeleton regulation are worst affected in its absence (Figures 6A,B) (detailed shown in the Supplementary Tables 2, 3).

### Immunoregulatory Effects of MFG-E8 Downregulation in BuMECs

We next set forth to determine the immunological behavior of the MFG-E8 associated protein, we mapped the proteome against the immune pathways and identified the B cells/T cells activation, lymphocytes activation, NK cell-mediated immunity, and leukocytes differentiation (Figure 6C). In correspondence with the multiple previous reports where MFG-E8 has been shown to elicit the immune response in terms of phagocytic activity in the MECs (Monks et al., 2002; Abrahams et al., 2004; Watson, 2006a, b; Stein et al., 2007; Watson and Kreuzaler, 2011). The detailed information about the identified GO terms is mentioned in the Supplementary Table 5 with fold enrichment and associated *p*-values and FDR.

Although MFG-E8 associated genome-wide studies were previously performed but only at the transcriptomics level (Sugano et al., 2011) and as per our knowledge, no MFG-E8 associated proteome-wide global ultra-deep proteomics study has been so far done. Moreover, looking into the importance of hundreds of genes target previously recognized (Sugano et al., 2011), and protein targets identified in our current study (Figure 4B and Supplementary Figure 5), point out several questions in regards to its diverse downstream associated signaling which remain unresolved. The critical prerequisite of MFG-E8 in basic organ developmental biology; such as mammary gland (Hu et al., 2009; Sugano et al., 2011), intestinal epithelium (Zhao et al., 2012), and lungs remodeling process (Aziz et al., 2015) further enforced to accomplish the comprehensive proteome analysis.

Interestingly, recent findings described that MFG-E8 derived short peptide, known as MSP68, showed immunomodulatory function in sepsis-injured tissue. These studies indicate the unique therapeutic property of MFG-E8 and its derived peptide for regulating the inhibition of the infiltrating neutrophils migration, especially in lungs sepsis (Hendricks et al., 2017; Hirano et al., 2017). In correspondence to these previous results, our proteomics data proves that the MFG-E8 mediated signals toward neutrophils activation and migration are severely compromised. The under enrichment of the immune-related proteins upon MFG-E8 knockdown and provide mechanistic insight into its downstream pathway.

### MFG-E8 Affects Signature Transcription Factors (TFs)

**FIGURE 7:**
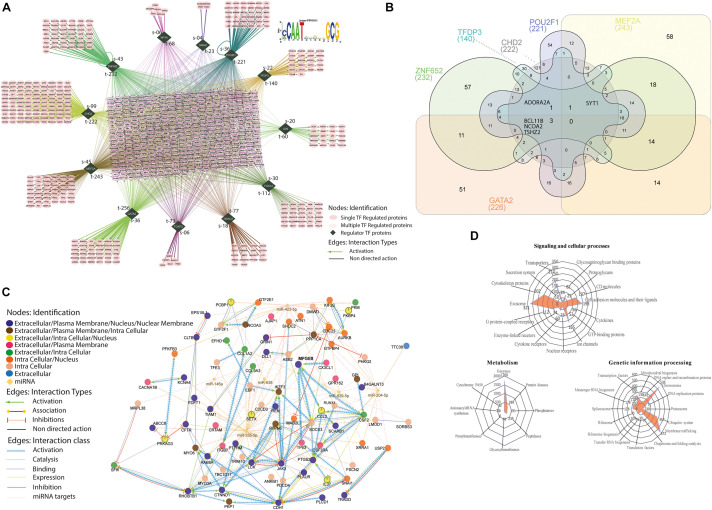
Transcription factor(s) associated with regulome. **(A)** All the identified significantly (*p* < 0.001) differentially expressed proteins with 1.5-fold change up and down were used, *n* = 938 proteins for the identification of associated TFs involved in the cell cycle regulation. The dark green rhombus shape nodes show the TFs, *S* defines the number of singly associated protein partners, *t* defines the total number of protein partners associated with individual TFs. Interactions are color-coded and detailed described in the bottom right downside. JASPAR based position-specific scoring matrices predicted the regulatory control sequences that represent the binding specificity of TFs. **(B)** Venn Diagram. For the comparison of six TFs and their identified number of target proteins. It determined the common targets proteins associated with six TFs (GATA2, ZNF652, TFDP3, CHD2, POU2F1, and MEF2A), the total of targets in individual TF is reported below in round bracket. Inside the Venn diagram, the number reports the common proteins. **(C)** A directed network of MFGE8–miRNA interactome. The *in silico* based network analysis of directly interacted miRNAs and their associated proteins partners, which was also identified as significantly (*p* < 0.001) differentially expressed in MFGE8 KD proteome. See also Supplementary Figure 5 for detailed analysis. **(D)** Radar plot. The identified proteins were mapped against the KEGG database for the determination of redundant involvement in molecular signaling and cellular processes, metabolic process, and genetic information processing. All the mapped proteins in the specific process were plotted in three different radar plots and indicated with the number of proteins determined.

Around 45% of the total profiled proteins belonged to the nucleus (Figure 4A). We determined the MFG-E8-dependent signature TFs with a cutoff of 1.5-fold up/down-regulated differentially expressed MEC proteome (*n* = 938 proteins) with significant *p*-value < 0.001) using a computational regulatory analysis with iRegulon (Janky et al., 2014). The analysis resulted in the identification of 12 TFs, which regulated 806 DEPs out of 938 identified proteins in the nucleus, as shown in the form of the interaction map (Figure 7A). The identified TFs and their targets includes ZNF652 (232), CHD2 (222), MEF2A (243), GATA2 (256), FOXF2 (73), HDAC2 (77), FOXA1 (112), JUN (60), TFDP3 (140), POU2F1 (221), NFYA (68), and SNAI2 (23). We identified a common motif of C/ACAATXXXXGCG on the target genome for binding with these TFs, as revealed by the analysis on the JASPAR database for transcription regulatory region DNA binding (GO: 0044212, *p*-value-0.0022).

The affected proteins with these TFs further narrowed down to control cell cycle regulation and maintenance (GO: 0007050, cell cycle arrest, *p*-value-0.004; GO: 0051301, cell division, *p*-value-0.021). Secondly, we discover all the targets were identified to be in direct connection with the individual TF; instead, a large portion was synergistically regulated, and extensive overlap resulted in the formation of the large regulon in the center of the interactome (Figure 7A). Particularly, among the 226 targets proteins that were activated downstream to GATA2, we found a combination of multiple TFs controlled 77.5% of the total targets. However, interestingly only three proteins (BCL11B, NCOA2 TSHZ2) were associated with all the TFs, and 51 proteins were associated with the only GATA2, as shown in the logic diagram (Figure 7A). The same is the case with MEF2A (243 total, 76.2% commonly regulated, 58 unique targets), POU2F1 (221 total, 75.6% commonly regulated, 54 unique targets), CHD2 (222 total, 45.5% commonly regulated, 121 unique targets), TFDP3 (140 total, 78.6% commonly regulated, 30 unique targets), and ZNF652 (232 total, 75.5% commonly regulated, 57 unique targets) (see Supplementary Table 7 for extensive details of the targets). These secondary regulons present the extensive overlap with the primary TFs regulon, intimating that these combinational functions of TFs are the leading contributors in gene regulation downstream to MFG-E8 (Figure 7B). The sharing of collective targets (more than 70% of each regulon) with the MFG-E8 regulon symbolizes the prevailing co-factorship among these TFs, something that has been described previously for individual TF, although not on such a lengthened order for the cell cycle proliferation. In extension, all of the TFs shown are implicated in cell cycle progression that is bona fide regulators of cell growth progression (Kumar et al., 2006; Koga et al., 2007; Qiao et al., 2007; Semba et al., 2017; Tang et al., 2018; Wang et al., 2018).

### Exploration of the Mechanism of MFG-E8 for Downstream Signaling

Next, we sought to determine the impact of MFG-E8 mediated signaling on cellular outcomes. For that, we also considered the possibility of a critical confounding variable of miRNA for the regulation and downstream signaling of MFG-E8 (Figure 7B). The combination of databases such as GeneMANIA, Reactome, CluePedia showed the activation of MFG-E8 through CX3CL1, TP63, and CSF2 in identified DEPs. The inferred directed network also indicates that MFG-E8 leads to activation of two proteins SOCS3 and CCL2, along with six miRNAs (miR-423-5p, miR-638, miR-939-5p, miR-204-5p, miR146a, and miR-335-5p) in the direct connection (Supplementary Figures 7, 8). The miR-423-5p and miR-638 were identified for autophagy promotion, while miR-204p and miR 335-5p were previously reported in invasion and metastasis suppression. The interaction is expanded by the finding of associated proteins from DEPs with miRNAs and MFG-E8 collectively and divided it into four underlying strata that are extracellular, plasma membrane, intracellular, and nucleus associated. However, the network is described with a particular interaction type for all diverse associations such as activation, association, inhibition, catalysis, binding, and expression indicated with an individual variety of edges (Figures 7B–D). It helped us to identify the specific molecules regulating cellular growth via down-regulated MFG-E8 such as AURKB, TRADD, SNAI1, USP2, and KIF2C. Figure 5 describes the illustrated signaling.

**FIGURE 8:**
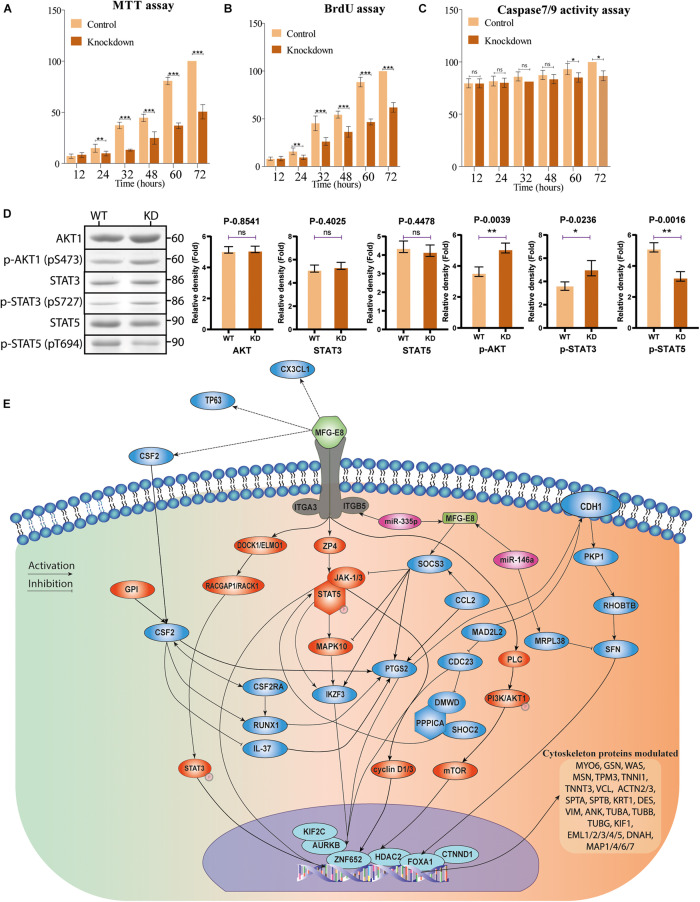
Multiple assays for validation. **(A)** MTT assay. The cell proliferation MTT assay for the control and MFG-E8 KD BuMEC cells were performed. All experiments were performed six times points at 12-h intervals up to 72 h of culture. The values were calculated using a non-parametric *t*-test, and the error bar is shown with a standard error of the mean (SEM). **p < 0.01, ***p < 0.001. **(B)** BrdU assay. The cell proliferation BrdU assay was assessed for the proliferation of the control and MFG-E8 KD BuMEC cells on every 12-h interval up to 72 h of culture by BrdU labeling. The values were calculated using a non-parametric *t*-test, and the error bar is shown with the standard error of the mean (SEM). **p < 0.01, ***p < 0.001. **(C)** Caspase-3/7 assay. Caspase-3/7 activities of the control and MFG-E8 KD BuMEC cells were measured by Caspase-Glo 3/7 activity assay kit. The cells were seeded into 96-well plates and incubated for 12 to 72 h with an interval of 12 h each. The Caspase-Glo 3/7 reagent was added and incubated for another 3 h. Fluorescence intensities of the wells were then measured using excitation wavelength 492 nm and emission wavelength 535 nm. The caspase activities of the normal WT BuMEC cells were compared to transfected KD BuMEC cells. All experiments were performed six times with almost similar results, and values were calculated using a non-parametric *t*-test, and the error bar is shown with the standard error of the mean (SEM). *p < 0.05, **p < 0.005, ***p < 0.001 and ns: non-significant. **(D)** Western blot analysis of scrambled shRNA WT and KD cells for AKT, STAT3 and STAT5 proteins with respective phosphoprotein antibody counterpart. Bar graph represent ± SD from three replicate. *p < 0.05, **p < 0.005, ***p < 0.001 and ns, non-significant. **(E)** Schematic representation for the signaling regulated through MFGE8 protein in mammary epithelial cells.

### Determination of Cell Proliferation and Caspase Activity in MFG-E8 KD_MEC Cells

The downregulation of MFG-E8 protein resulted in the slow growth of the cells in MFG-E8 KD_MEC cells (Figures 8A–C). MTT assay results indicated that there was a decline in the MTT reductive capacity in MFG-E8 KD_MEC cells over different time points in culture (12, 24, 32, 48, 60, and 72 h) (Figure 8A), which was also validated by BrdU assay (Figure 8B). The cell growth rate was relatively equal for 12 and 24 h (non-significant *p* > 0.05). In comparison, it continuously declined from 32 to 72 h onward to nearly half with significant *p* > 0.001 (Figure 8B). Interestingly, these cellular proliferation assay showed a high growth rate of control cells in comparison with the MFG-E8 KD_MEC cells, especially at 72 h of cell incubation. To check whether MFG-E8 knockdown is leading to apoptosis in MEC cells, we performed the caspase 3/7 activity assay for all the same 6-time intervals (Figure 8C). Caspase 3/7 activity results were identified as non-significant changes for nearly all time points, suggesting that the decrease in the proliferation shown in cell growth is because of knockdown of MFG-E8 and not due to the induction of apoptosis. The findings were also confirmed through the presence of 11 different anti-apoptotic class proteins or lower expression of caspase proteins fold change was perceived (Supplementary Table 9). The results point out that MFG-E8 knockdown does not lead to caspases mediated apoptotic pathway. Similarly, MFG-E8 downregulation remarkably repressed wound healing and cell migration which is also highlighted in the enrichment analysis GO: 0016477, GO: 0043536: cell migration (*p*-value 0.000104) were validated by the migration assay (*P* < value 0.0001) WT versus WT + HA14-1/KD/KD + HA14-1 proves our finding of proteomics-based pathway analysis data.

Dynamics of proteins phosphorylation majorly regulate the cellular signaling. From the network analysis, we selected three key TFs (AKT1, STAT3, and STAT5) and counterpart phosphoproteins for western blot analysis. We identified the non-significant changes in the non-phosphorylated AKT1, STAT3 and STAT5 proteins. However, significant upregulation of p-AKT (p-S473) and p-STAT3 (pS727) in KD cells were observed, but surprisingly, p-STAT5 (p-T694) was decreased in KD cells in comparison to WT cells (Figure 8D). The results are in correspondence to the proteomics data. Collectively, these data support that the dynamic phosphorylation event is more critical for the regulation of downstream signaling (Figure 8E).

## Discussion

The current study determined the signaling of MFG-E8 in MECs and its involvement in cellular proliferation and homeostasis. MFG-E8 protein regulates apoptotic-based cell death, especially the clearance of dying epithelial cells (Clarkson et al., 2004; Stein et al., 2004). The accumulation of milk at the end of the lactation stage in mammary glands triggers the death of excessive epithelial cells. These cells are promptly removed by neighboring epithelial cells and by infiltrating phagocytes with the help of MFG-E8 protein to ensure that the gland returns to its pre-pregnant state (Ensslin and Shur, 2007; Monks et al., 2008; Watson and Kreuzaler, 2011; Sumbal et al., 2020). Therefore, we focused on determining the pathways associated with MFG-E8 protein in MEC proliferation and oncogenesis processes.

Initially, our global sequence analysis across the species showed the involvement of MFGE8 protein in vesicle trafficking and membrane-associated ion channels, which functions for intracellular transportation (Peng and Elkon, 2011). Further, we found that the downregulation of MFG-E8 *in vitro* resulted in impaired epithelial morphology and decreased cell growth. The reduced cell growth rate is confirmed by cell growth confluence assay, wound healing and migration assay. The reason could be the lower expression of MFG-E8 is associated with a high level of cyclin-dependent kinase 5 activator protein (CDK5R2, FC-2), resulting in reduced cell growth. A similar observation was seen where MFG-E8 silencing in 4T1 cells reduces the cyclin protein expression level (Carrascosa et al., 2012). Nonetheless, MFG-E8 overexpression in MEC showed aggressive tumor growth *in vivo* (34).

Our cell cycle analysis in WT versus KD cells showed the increased cell cycle progression in KD cells. The reason could be the differential expression of cyclin D1, which regulates cell cycle progression during the G1/S transition. Its expression is increased at the activation of oncogenes such as Ras or Neu and found to be up-regulated in breast cancer (Lee and Yang, 2003; Roy and Thompson, 2006). The transgenic mice overexpressing the cyclin D1 protein in the mammary gland resulted in hyperplasia and further led to neoplastic transformation (Wang et al., 1994). However, knockout in transgenic mice producing oncogenic protein Ras prevents mammary tumor development (Yu et al., 2001). Down-regulation of MFG-E8 resulting in low expression of cyclin D1 in MEC suggests that it is a valuable player involved in the initiation of MEC transformation, pushing it toward slowing down the cell growth; that is an essential requirement during the involution process.

Furthermore, in our iTRAQ based quantitative proteomic dataset, the network analysis reveals direct molecular linkage between knocked down state MFG-E8 activating ZP4 and STAT3/5 proteins. The STAT family proteins are orchestrators of mammary gland remodeling via cell proliferation and involution (Resemann et al., 2014). These TFs are present in the downstream signaling through various receptors, including cytokines receptors, growth factor receptors, and G-protein-coupled receptors. Here, we found that the ZP4 is physically interacting with JAK1/3-STAT5 and ITGA3/ITGB5 complex. Previously, the immunoprecipitation based results validate our findings, where zona pellucida-like domain-containing protein 1 (CUZD1), a similar protein like ZP4 in MECs, was found physically associated JAK-STAT5 complex (Mapes et al., 2017). The amplification of the CUZD1/ZP4 expression is associated with increased JAK1/JAK2/STAT5 signaling, which translocates the STAT5 into the nucleus, but the mechanism is poorly understood (Mapes et al., 2017). We determined that ZP4 potentiates JAK/STAT signaling downstream of MFG-E8 by mediating ITGA3/ITGB5 receptor activation by our bioinformatics data bridging an adaptor that assists in the recruitment of STAT5 to the stabilizing JAK1/3-STAT5 complex. The available literature about the roles of the effector proteins is the primary precedence of this hypothesis that alters signaling through this complex (Rawlings et al., 2004; Seif et al., 2017). An interesting example is a c-Src protein, which has been shown to propagate PRL initiated JAK/STAT signaling in healthy mammary tissue (García-Martínez et al., 2010). Likewise, STAT5 signaling is inhibited by the caveolin-1 (Cav-1) pathway by competitively binding to the domain of JAK2 tyrosine kinase, preventing subsequent activation of STAT5 (Park et al., 2002).

Another molecule regulated by MFG-E8 downstream signaling is DOCK1 resulted in the activation of RACGAP1 (FC-0.8)/RACK1 (FC-0.54) complex by STAT3 phosphorylation on Y705 (Tonozuka et al., 2004) and further transfer to the nucleus to act as a nuclear chaperone (Kawashima et al., 2009). The activation of Rac1 signaling supports the expression level of IL-6 production (Arulanandam et al., 2009), which is complementary to our results were down-regulation of this pathway results in the decreased expression of interleukins (with respective FCs; IL-6, IL-9, IL-16 0.52, IL-18 0.47, and IL-21). Previously, it was observed that the absence of DOCK1 in small mammary gland neoplasia reduced the STAT3 activation (Laurin et al., 2013). Here, we showed that the expression of STAT3 is diminished in the absence of MFG-E8 along with RAC1 and DOCK1. These results suggest that DOCK1 is a key GEF activating Rac1 complex and initiates the STAT3 activation mutually in mammary gland involution and breast cancer. It will be interesting to investigate the molecular intermediates of the RACGAP1/RACK1 complex *in vivo* to connect to STAT3 signaling pathway mammary gland developmental physiology as the down-regulation of this signaling resulted in the delays of the proliferation of MEC growth (Figure 8E).

On the other hand, we revealed that MFG-E8 mediated DOCK1/Rac1 signaling is essential for non-professional epithelial phagocytes to endorse the engulfment of apoptotic cells. This work is the first demonstration to address possible global conservation of the function of MFG-E8 mediated DOCK1/RAC1 signaling in dead cells clearance. The DOCK1- binding partner ELMO1 was formerly revealed to operate downstream to the PtdSer receptor BAIAP3 (a form of Bai1) to support the removal of dead cells. An *in vivo* study of ELMO1 mutant had shown a defect in removing apoptotic germ cells in the testis (Park et al., 2007; Elliott et al., 2010). Based on these results, we further confirm that BAIAP3 is necessarily required by epithelial cells to promote activation of the MFG-E8/DOCK1/ELMO1/RAC1 component to stimulate the elimination of dead MECs with respect to mammary gland involution. Simultaneously, the other phagocytic receptors, for example, ITGA3/ITGB5 or Tyro3, Axl, or MerTK, are equally vital to clear dying cells through DOCK1/RAC1 signaling. We reason that MFG-E8 mediated signaling is responsible for the activation of the DOCK1/RAC pathway, mainly required for the clearance of dying MECs.

The importance of the PI3K/AKT pathway is typically indispensable, and it plays a crucial role in the process of cell proliferation, survival, motility, secretion, apoptosis, and metabolism (Freudlsperger et al., 2011; Kamal et al., 2016). The activation of the AKT pathway supports cell proliferation, but only limited reports have clearly explained the relationship between MFG-E8 and PI3K/Akt signaling (Neutzner et al., 2007; Jinushi et al., 2008). Our results indicate that downregulation of MFG-E8 leads to decreased expression of multiple PLC proteoforms mediated PI3K/AKT (FC 0.70) signaling via mTOR modulating the expression of TFs which resulted in the slow proliferation of the MEC cells. The results from the MTT, BrdU, and cell growth confluence assay suggested that the down-regulation of MFG-E8 reduces the cell proliferation rate and does not induce apoptosis, as confirmed by the caspase 3/7 activity assay. Our results of shRNA-based knockdown of the MFG-E8 in MECs cells are an excellent reference for future clinical trials. It was previously shown that preventing MFG-E8 signaling via anti- MFG-E8 antibodies caused the regression of experimental breast cancers (Ceriani et al., 1987). Concomitantly, using this approach, synergistically and traditional chemotherapy also showed reduced experimental colon carcinomas, melanomas, and lymphomas (Jinushi et al., 2009). MFG-E8 silenced triple-negative breast cancer cells showed higher sensitivity to cisplatin treatment (Yang et al., 2011). In this regards, collectively present results and published evidence, promoting the notion that MFG-E8 is a constitutive molecule essential for cellular homeostasis. Its expression pattern changes during mammary gland development to restore the molecular balance. This study also highlights that combinatorial therapies combined with the blocking of MFG-E8 may prove sufficiently effective outcomes in breast cancer treatment.

## Conclusion

In this study, we utilized the benefits of a global proteomic workflow for measuring the crucial low abundant endogenous TFs network of signaling involved in cellular homeostasis downstream to MFG-E8 knockdown. Specifically, we observed that the absence of MFG-E8 suppresses cell growth, alters cellular morphology, and activates the immune function. Necessarily, suppression of MFG-E8 results in reduced growth of MECs by an autocrine/paracrine mechanism that may imply the induction of TFs such as cyclins D1, FOXA1, ZNF652, AURKB and many others for modulating the cytoskeleton-associated proteins and cell cycle regulators. The significance of MFG-E8 signaling concerning mammary gland developmental remodeling and disease is indisputable, and this study, through proteomic examination, has underscored its importance in cellular homeostasis. Finally, we anticipate that our comprehensive datasets will serve as a valuable foundation for further validation and mechanistic exposition of many novel proteins that are involved in MFG-E8 meditated signaling in the mammary gland.

## Data Availability Statement

The datasets presented in this study can be found in online repositories. The names of the repository/repositories and accession number(s) can be found in the article/Supplementary Material.

## Author Contributions

SA and PS designed and performed the experiments, conducted data analysis, and wrote the manuscript. AV helped in initializing the experiments. SK helped in the data analysis. AM supervised the whole project and reviewed the manuscript. All authors contributed to the article and approved the submitted version.

## Conflict of Interest

The authors declare that the research was conducted in the absence of any commercial or financial relationships that could be construed as a potential conflict of interest.

## Footnotes

1 http://www.genome.jp/kegg/mapper.html

## Supplementary Material

Supplementary Figure 1 Full-length multiple sequence alignment of all 35 organisms with %Identity wise color code. Total numbers of amino acid residue present in the individual proteins sequence in all organisms are reported with the name. The consensus sequence logo was shown in the downside of the alignment, and the conserved nature was depicted using the size of the amino acid single alphabet symbol. Conservation and quality are reported using histograms. The highest histogram defines high conservation, and small histograms are low conservation.

Supplementary Figure 2 Statistically significantly enriched motifs identified using multiple sequence alignment (MSA) of MFGE8 proteins retracted from Motif-X analysis. Motif-X online tool was used to identify the motifs of six amino acid residues for the identification of two specific post-translational modification (PTM) motif sequences. The amino acid residues are STY for phosphorylation and SWRN for glycosylation. The parameters used for the Motif-X algorithm were occurrences, 10; significance level, 0.00001.

Supplementary Figure 3 Multi scatters plot. The graph represents the correlation of all the samples to all for the identification of the variable dependency.

Supplementary Figure 4 Correlation and density plot. The iTRAQ intensities obtained after the MS data extraction was plotted to find the correlation among the replicates and between the conditions. The Pearson correlation *R* values were determined and reported in the graph. The blue color defines the high density of the spectra for the proteins and while the green color shows low, dense proteins.

Supplementary Figure 5 The relative abundance of various signaling pathways based on DEPs iTRAQ data using the KEGG database (shown as that of KD versus WT cells). Gene Ontology is represented as fold enrichment. Probability was determined using a binomial statistic for the false discovery rate (FDR) and a *p*-value cutoff of % 0.05 significance level.

Supplementary Figure 6 FACS analysis. Analysis of the BuMEC cell population before and after a knockdown by induces cell cycle arrest. **(A)** Flow cytometry analysis of the MFG-E8 KD cell cycle reveals that knockdown induces cell cycle arrest at the G0-G1 phase: 32.62%, G2-M phase: 20.18%, and S phase:47.20%. **(B)** While the analysis of the MFG-E8 WT cell cycle reveals induces cell cycle arrest at the G0-G1 phase: 18.42%, G2-M phase: 16.10%, and S phase: 65.85%.

Supplementary Figure 7 Protein miRNA network of MFGE8–miRNA interactome. The *in silico* based network analysis of directly interacted miRNAs and their associated proteins partners, which was also identified as significantly (*p* < 0.001) differentially expressed in MFGE8 KD proteome. Circular nodes denote the proteins, diamond-shaped nodes are regulation miRNA partners. Dotted lined denotes the edges for miRNA to protein interactions, whereas the broad green line denotes the miRNA targets.

Supplementary Table 1 Total combined identified protein and peptide list together with the identification of all ontological information associated in the Uniprot database.

Supplementary Table 2 Classification of BuMEC proteome using PANTHER (Protein ANalysis THrough Evolutionary Relationships) curated biological database for protein families and their functionally related subfamilies to classify and identify the function of proteins.

Supplementary Table 3 The resulted list of DAVID (the database for annotation, visualization, and integrated discovery) Functional Annotation Tool and typical protein-term enrichment analysis.

Supplementary Table 4 KEGG BRITE annotation of MFGE8 linked differentially expressed proteins.

Supplementary Table 5 Information from the ngLOC prediction of proteins classified in to different categories of cellular organelles.

Supplementary Table 6 Information from string enrichment analysis for all the associated proteins involved in network construction.

Supplementary Table 7 The determined information for the targets of TFs identified associated proteins in MFGE8 downstream signaling.

Supplementary Table 8 Information on miRNA target proteins and interaction analysis for all the associated proteins involved in network construction.

Supplementary Table 9 The information for the identified DEPs associated with apoptosis activity in the proteomics dataset.

## Abbreviations

Q-TOF: quadrupole time-of-flight
MFG-E8: milk fat globule-EGF factor 8 protein
MECs: mammary epithelial cells
MFGM: milk fat globules membrane
SNP: single nucleotide polymorphism
SLE: systemic lupus erythematosus
DiGE: difference gel electrophoresis
TFs: transcription factors
BoMac: the bovine macrophage
BuMEC: buffalo mammary epithelial cell
KD_MEC: KD BuMEC_MFG-E8 cell line
GFP: green fluorescent protein
DEPs: differentially expressed proteins
GEF: guanine nucleotide exchange factor.
