# Supplementary material for: Transcriptional Repression of MFG-E8 Causes Disturbance in the Homeostasis of Cell Cycle Through DOCK/ZP4/STAT Signaling in Buffalo Mammary Epithelial Cells
Source: Front Cell Dev Biol. 2021 Apr 1;9:568660. doi: 10.3389/fcell.2021.568660 (PMC8047144; doi:10.3389/fcell.2021.568660)
Supplement: Supplementary Figure 1 — Full-length multiple sequence alignment of all 35 organisms with %Identity wise color code. Total numbers of amino acid residue present in the individual proteins sequence in all organisms are reported with the name. The consensus sequence logo was shown in the downside of the alignment, and the conserved nature was depicted using the size of the amino acid single alphabet symbol. Conservation and quality are reported using histograms. The highest histogram defines high conservation, and small histograms are low conservation. [file Presentation_1.PPTX]

## Slide 1
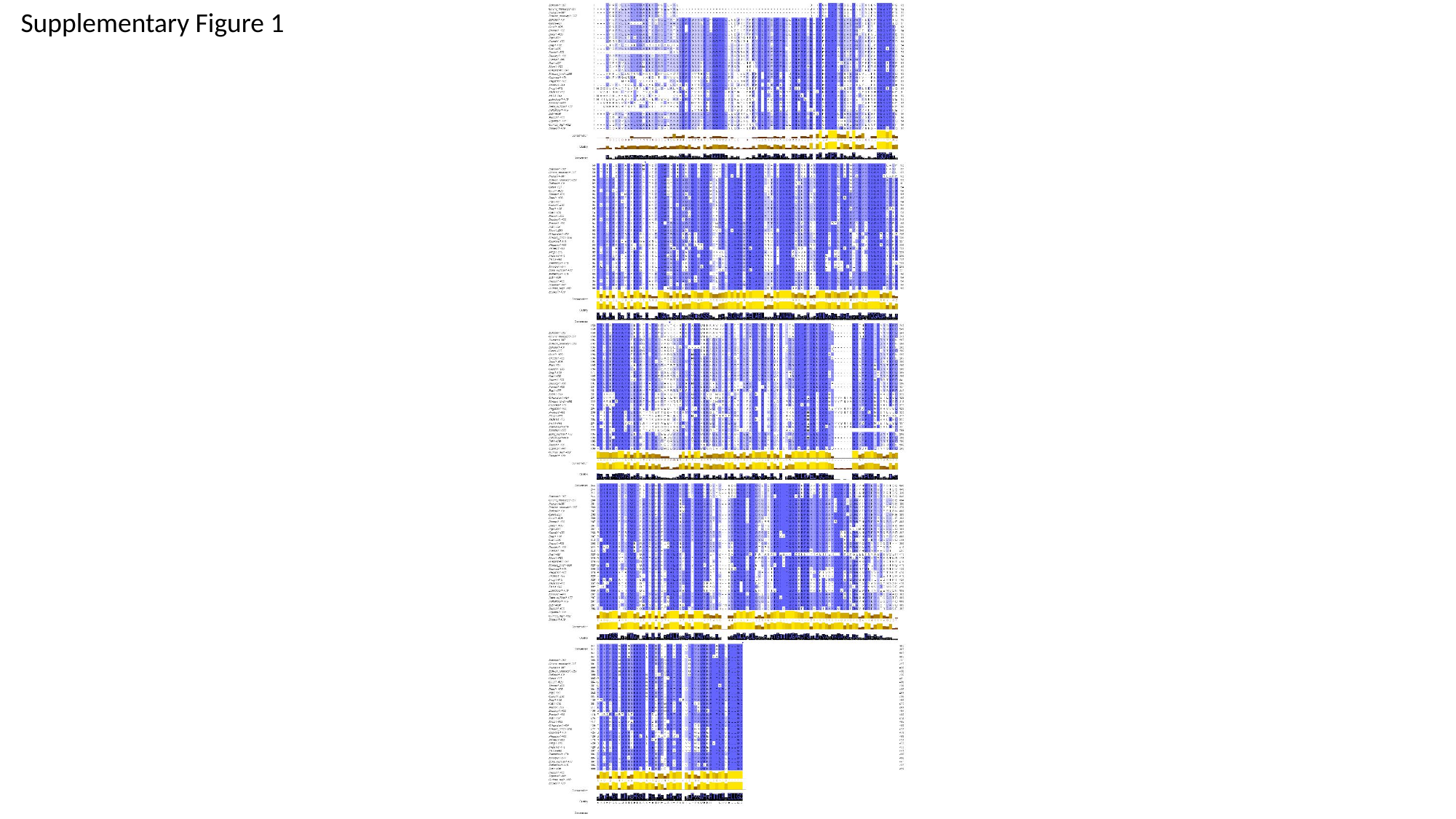

Supplementary Figure 1

## Slide 2
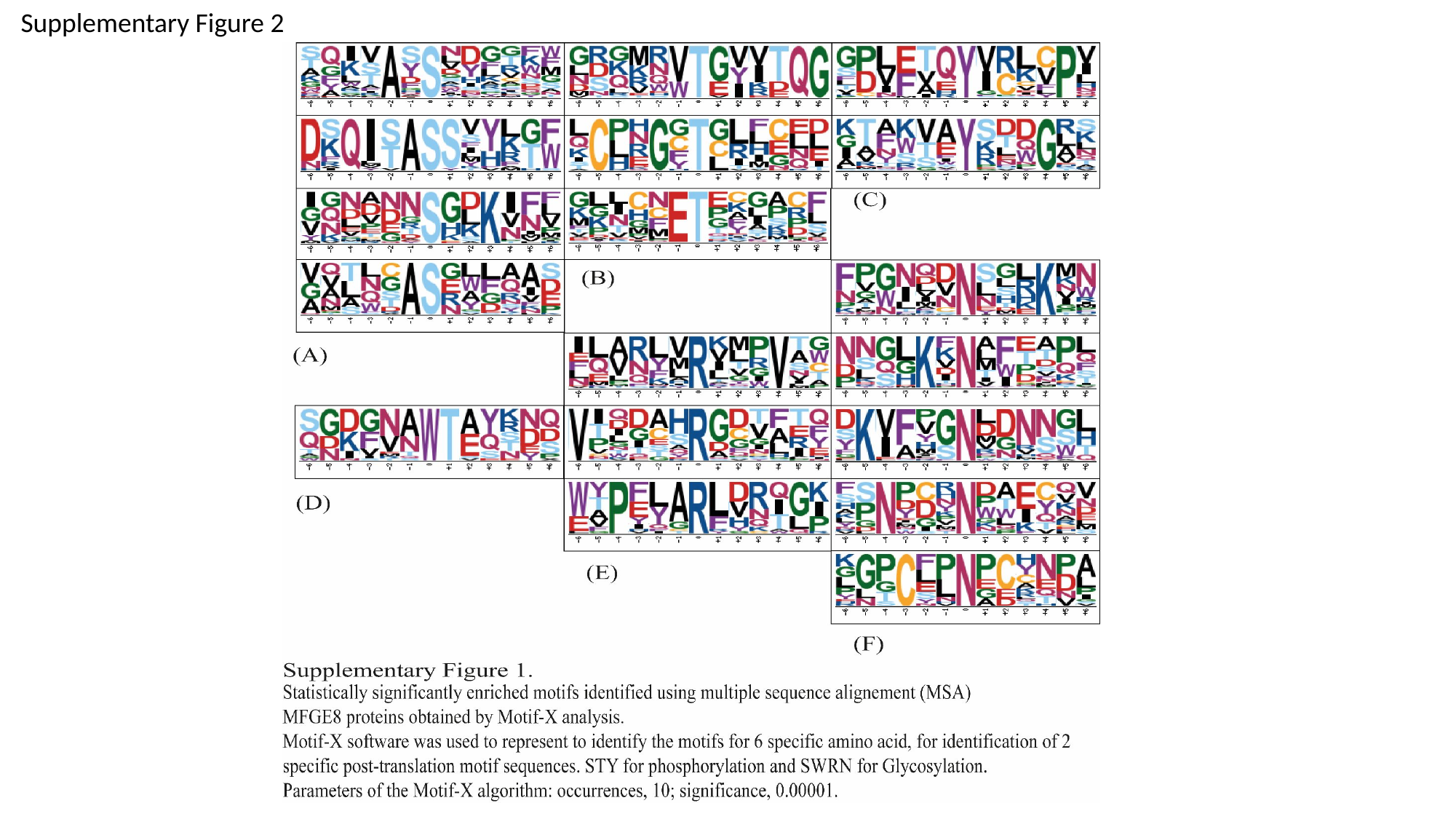

Supplementary Figure 2

## Slide 3
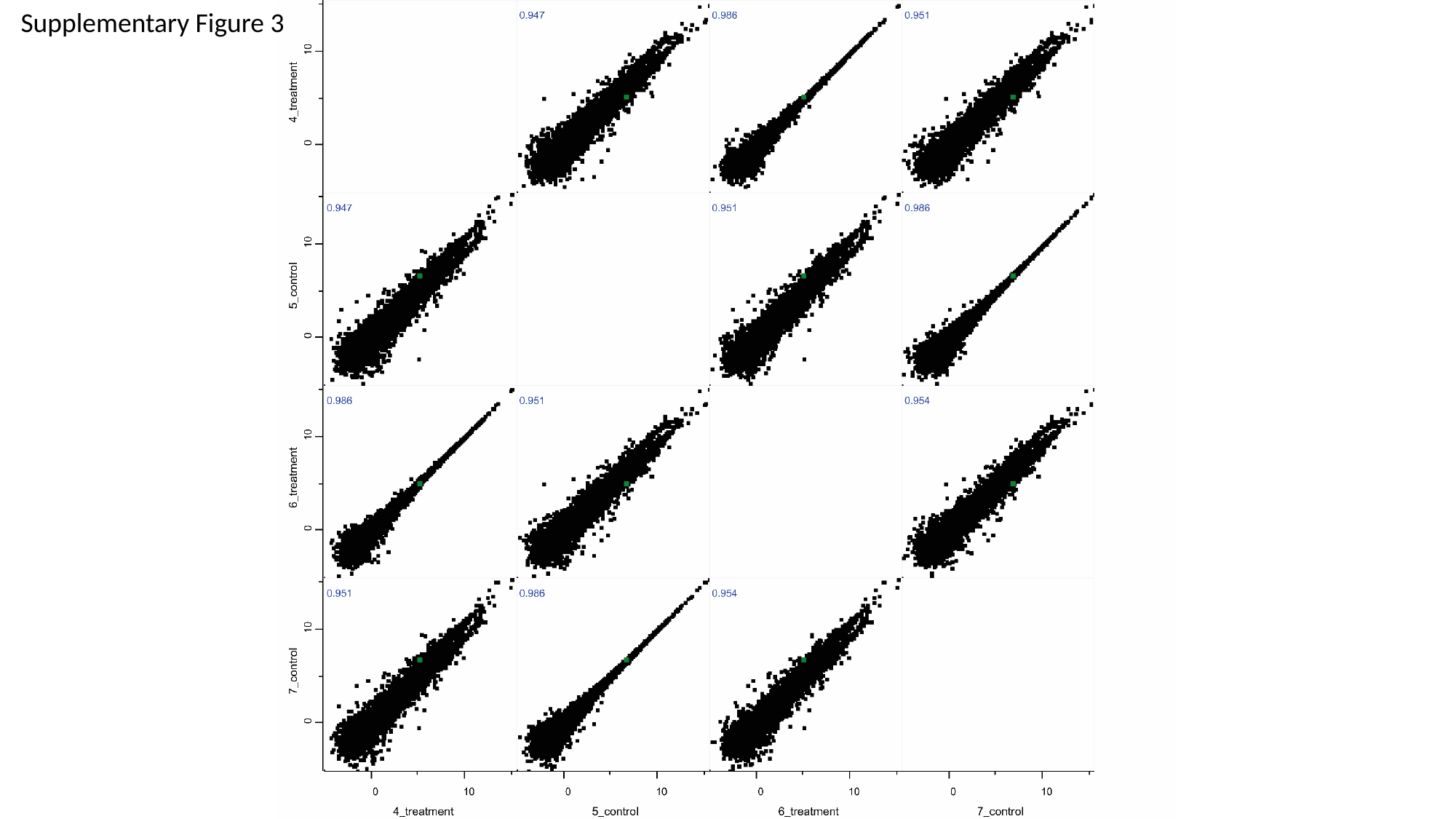

Supplementary Figure 3

## Slide 4
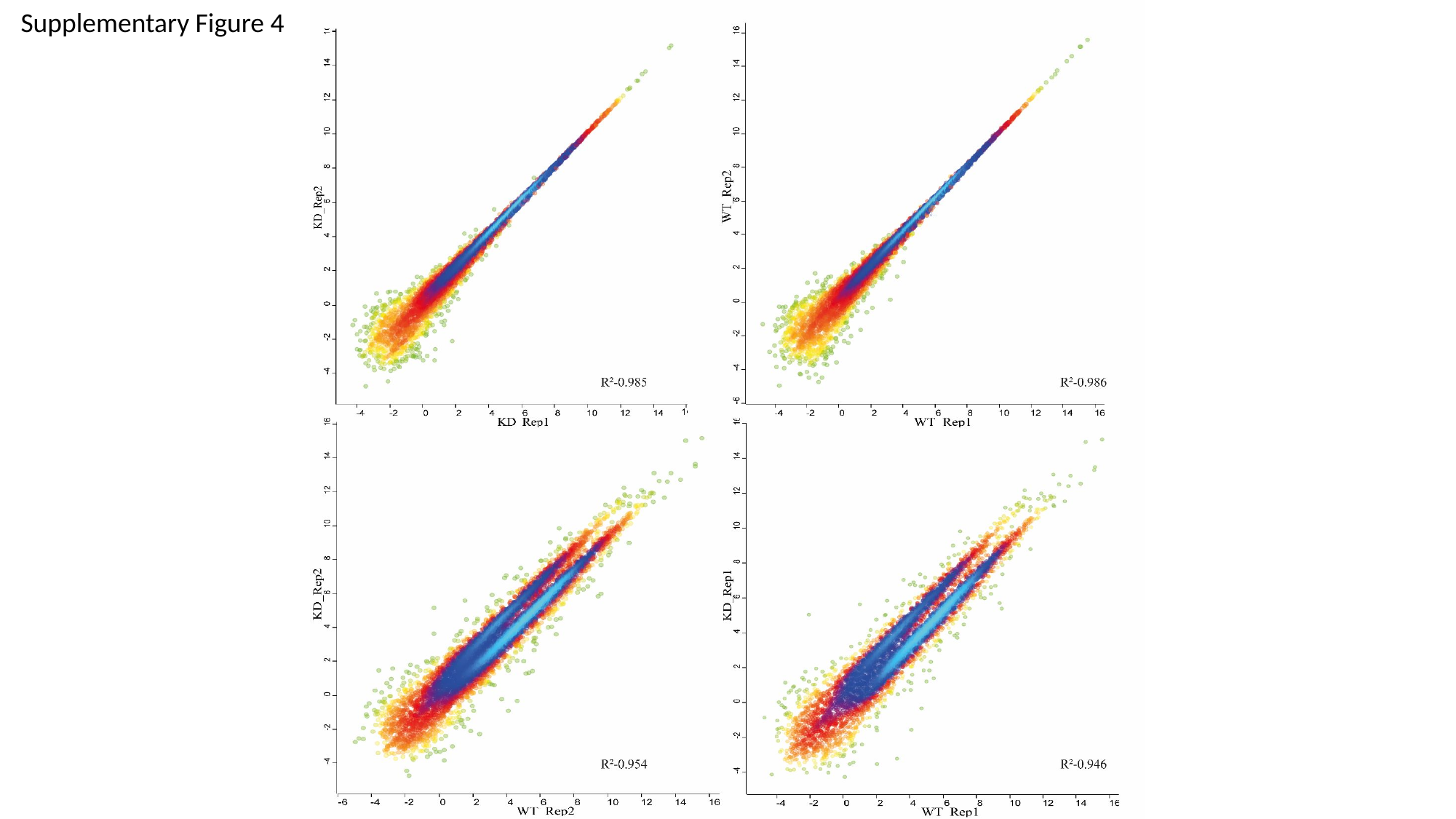

Supplementary Figure 4

## Slide 5
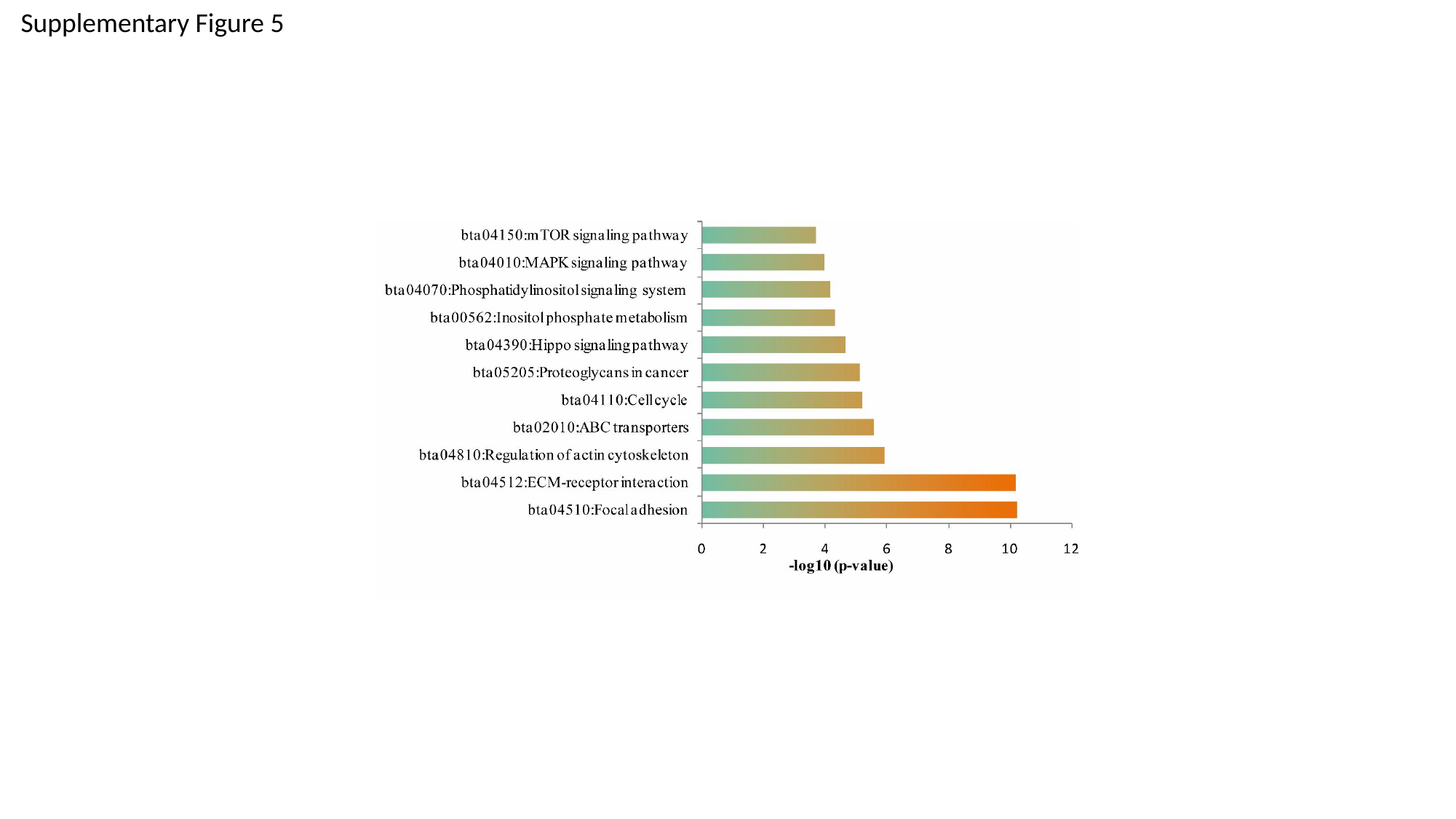

Supplementary Figure 5

## Slide 6
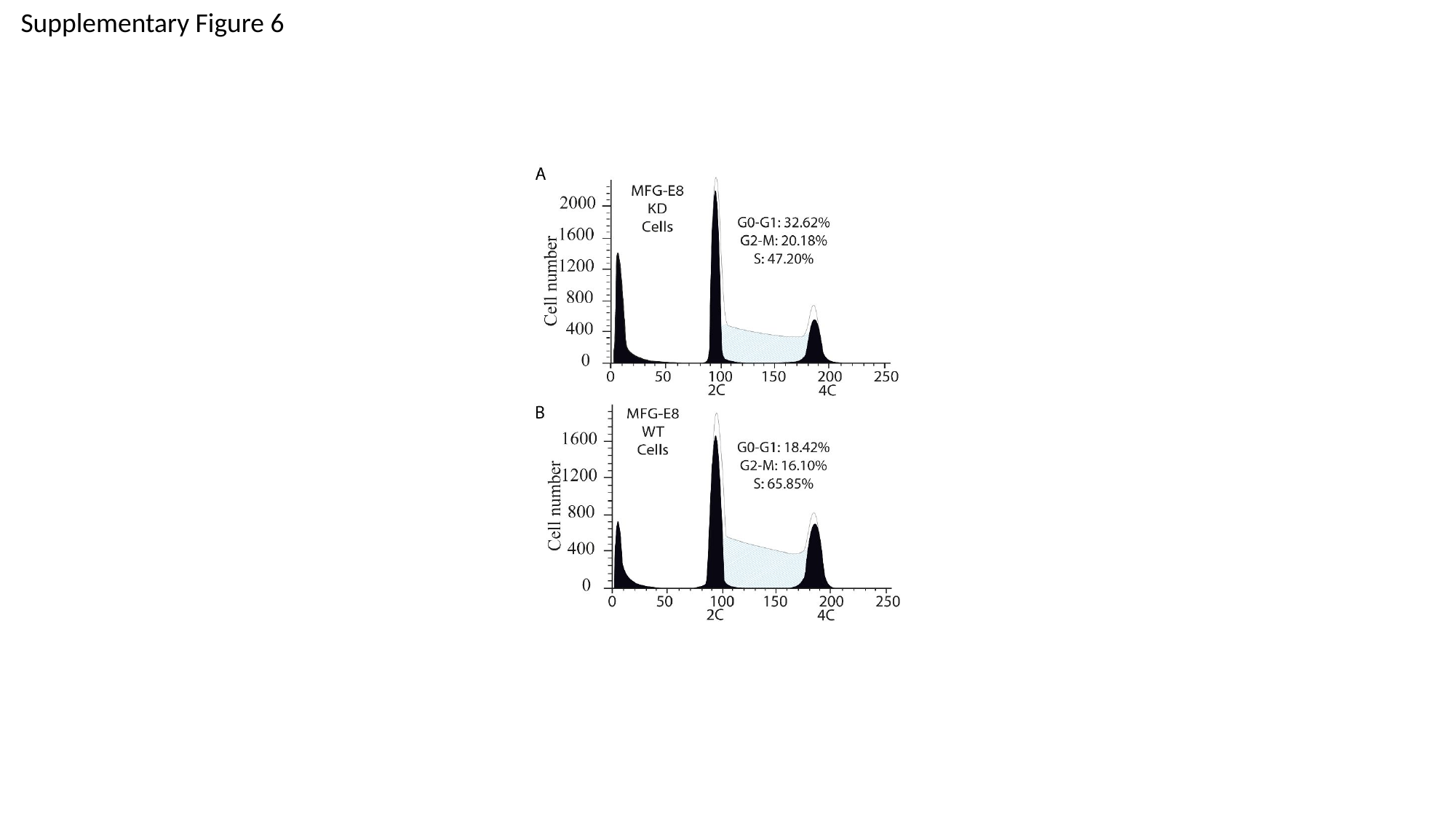

Supplementary Figure 6

## Slide 7
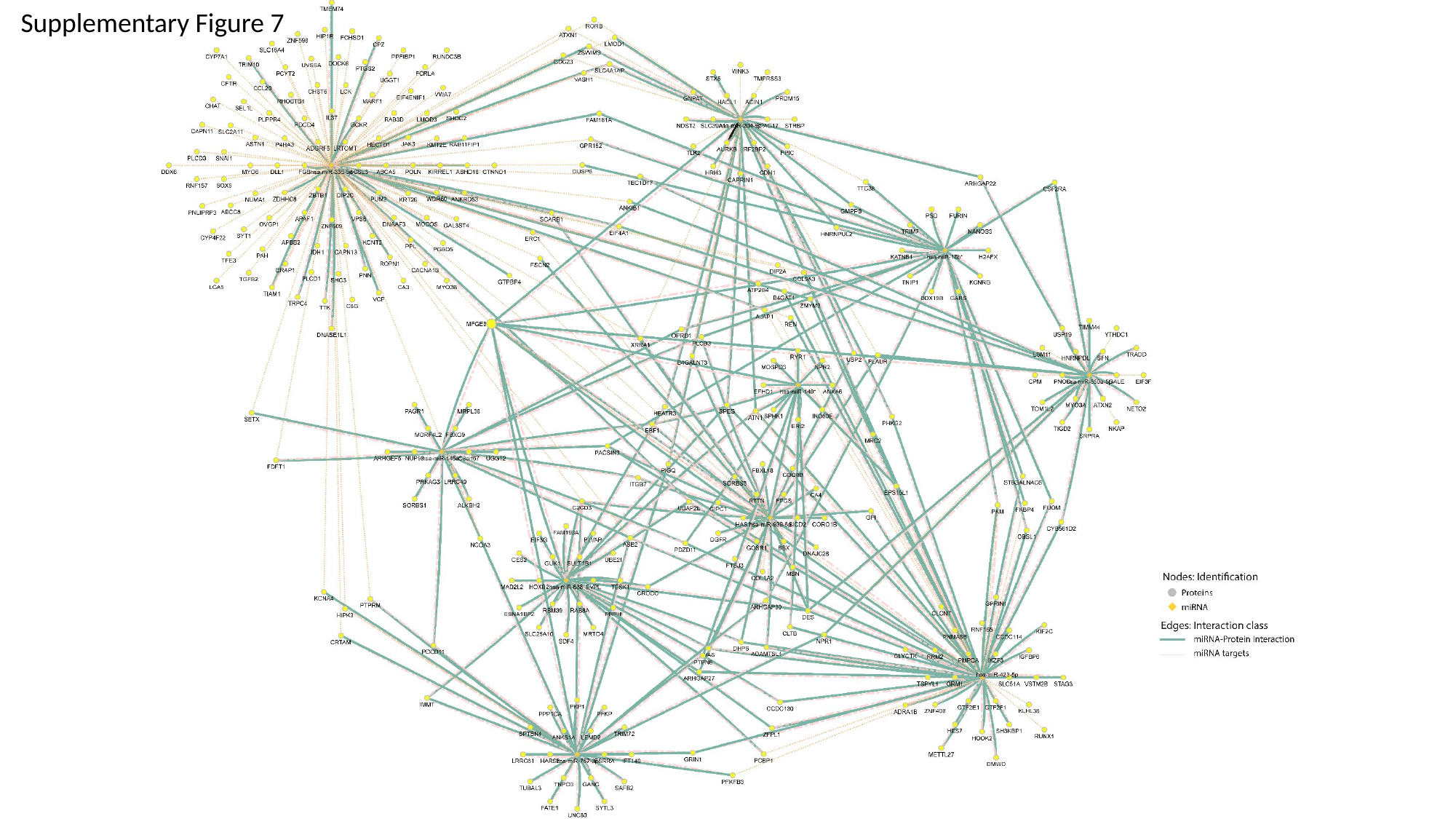

Supplementary Figure 7
